# Use and impact of risk-based eligibility models in low-dose computed tomography lung cancer screening: a systematic review

**DOI:** 10.3389/phrs.2026.1609133

**Published:** 2026-07-20

**Authors:** Veronika Elisabeth Mikl, Mohammad Azizzadeh, Marie-Kathrin Breyer, Kevin ten Haaf, Valentin Ritschl, Judit Simon, Tanja Stamm

**Affiliations:** 1 Doctoral Program Public Health, Institute of Outcomes Research, Center for Medical Data Science, Medical University of Vienna, Vienna, Austria; 2 Roche Austria GmbH, Vienna, Austria; 3 Ludwig Boltzmann Institute for Lung Health, Vienna, Austria; 4 Faculty of Medicine, Sigmund Freud Private University, Vienna, Austria; 5 Department of Respiratory and Pulmonary Diseases, Site Penzing of Clinic Ottakring, Vienna Healthcare Group, Vienna, Austria; 6 Department of Public Health, Erasmus MC, University Medical Center Rotterdam, Rotterdam, Netherlands; 7 Institute of Outcomes Research, Center for Medical Data Science, Medical University of Vienna, Vienna, Austria; 8 Ludwig Boltzmann Institute for Arthritis and Rehabilitation, Vienna, Austria; 9 Department of Health Economics, Center for Public Health, Medical University of Vienna, Vienna, Austria; 10 Department of Psychiatry, University of Oxford, Oxford, United Kingdom

**Keywords:** cost-effectiveness, early detection, eligibility criteria, low-dose computed tomography (LDCT), lung cancer screening

## Abstract

**Objectives:**

Low-dose computed tomography lung cancer screening (LDCT-LCS) significantly reduces mortality, yet identifying high-risk individuals while reducing over-screening remains challenging. Risk-based eligibility models are promising to optimize participant selection. Within the European context, we assessed the types, outcomes, and impact of these risk-based eligibility models for LDCT-LCS.

**Methods:**

We systematically reviewed prediction model studies (PROSPERO CRD42025648906) across EMBASE, MEDLINE, and Cochrane Central Register of Controlled Trials. We included original research on adults aged 18+ at risk for LC, excluding East-Asian populations. Study characteristics, model type, performance and outcomes were extracted for narrative synthesis.

**Results:**

The review included 46 articles (2003–2025), identifying 39 risk-prediction models. Models were primarily statistical (72%); PLCOm2012 was most frequent. Age (100%), smoking duration (91%), and intensity (72%) were the most common variables. Risk models improved eligibility and demonstrated cost-effectiveness over traditional criteria, though heterogeneity and population-specific calibration remain challenges.

**Conclusion:**

Risk-based eligibility models improve LDCT-LCS efficiency by enhancing detection rates and personalization. While PLCOm2012 is prominent, addressing model heterogeneity, ensuring population-specific validation, and calibration are crucial to optimize LDCT-LCS outcomes in Europe.

**Systematic Review Registration:**

Identifier CRD42025648906.

## Introduction

Lung cancer (LC) remains a significant global health burden and the leading cause of cancer-related mortality [1–3]. In 2023, LC accounted for 229,920 of 1.16 million cancer-related deaths in the European Union (ICD-10 C33-C34), representing almost one in five (19.8%) of all cancer fatalities [4]. Low-dose computed tomography (LDCT) lung cancer screening (LCS) significantly reduces LC-specific mortality, with landmark trials like the US-based NLST and the European NELSON study demonstrated mortality reductions of 20% (95% CI, 6.8 %–26.7%; p = 0.004) [5] and 24% (cumulative rate ratio of 0.76; 95% CI, 0.61–0.94; p = 0.01) [2] respectively. Consequently, authorities increasingly recommend the implementation of LDCT-LCS [6–9]. As of April 2025, 18 countries in Europe have initiated LCS implementation. The Czech Republic, Croatia, Poland, and the United Kingdom were the first European countries with national LDCT-LCS programs [10], Germany has followed in 2026 [11] and Austria is actively discussing implementation [12].

Despite LDCT-LCS benefits, accurately identifying high-risk population and reducing over-screening remains a challenge. Current LCS eligibility recommendations often rely solely on age and smoking history. While valid predictors, this restricted approach can miss high-risk individuals falling just outside standard thresholds, while over-screening those who meet the broad criteria but possess low actual risk. Risk-based eligibility models for LDCT-LCS, which integrate these foundational variables with a broader range of supplementary risk variables, can significantly optimize participant selection [13–15]. Although eleven countries have already integrated “risk-based eligibility modeling” into their LCS pilots [10], a comprehensive understanding of the most relevant risk variables, their real-world applicability, and the potential of new technologies remains crucial.

Therefore, this systematic review assesses the types and outcomes of risk-based eligibility prediction models for LDCT-LCS to inform policymakers, clinicians, and researchers—particularly within European healthcare systems discussing LCS implementation. Because models developed in North America and Australia represent some of the most extensively validated tools, studies evaluating these were intentionally included to benchmark their performance and assess their transferability compared to models developed for European populations. Specifically, this review seeks to identify these models, assess the characteristics of their eligible populations, and describe their impact on LDCT-LCS efficiency.

## Methods

We conducted a systematic review of prediction model studies to synthesize evidence on the types and outcomes of risk-based eligibility prediction models in LDCT-LCS. The protocol was prospectively registered with PROSPERO [16]. This review adheres to the TRIPOD SRMA Checklist [17] for reporting systematic reviews of prediction model studies, and the completed checklist specific to this article is provided in the Supplementary Material S1.

### PICO criteria and eligibility

This review focused on original research studies involving individuals aged ≥18 at risk for lung cancer. We excluded studies with pre-existing lung cancer diagnoses or those primarily on East-Asian populations, given documented differences in lung cancer aetiology, genetics, and progression that could limit risk model comparability [18, 19]. Our intervention of interest was LDCT-LCS utilizing risk-prediction models (e.g., PLCOm2012). Comparisons included no screening, alternative modalities like chest radiography, or LDCT-LCS based solely on age and smoking status. Outcomes examined encompassed process-related aspects (specific models, tools, parameters, eligibility criteria, screening intervals, uptake, adherence), performance outcomes (sensitivity, specificity, cases detected), health outcomes (mortality, survival, patient-reported outcomes like QALYs), and health economic outcomes (cost-effectiveness). The review included original quantitative full-text publications. Review articles, systematic reviews, and meta-analyses were screened to identify relevant original studies; qualitative studies were excluded.

### Search strategy

The review employed a systematic search strategy across three databases via OVID: EMBASE, MEDLINE, and the Cochrane Central Register of Controlled Trials. The search combined: “lung cancer,” “mass screening,” “low-dose computed tomography,” and “risk prediction.” Keywords were searched as free text and within EMTREE and MeSH subject headings. No language or date restrictions were applied, and no additional search filters were used. Search results were compiled, tabulated, and duplicates removed. Detailed search strategies for each database are in Supplementary Materials S2, S3.

### Article selection and data extraction

**FIGURE 1:**
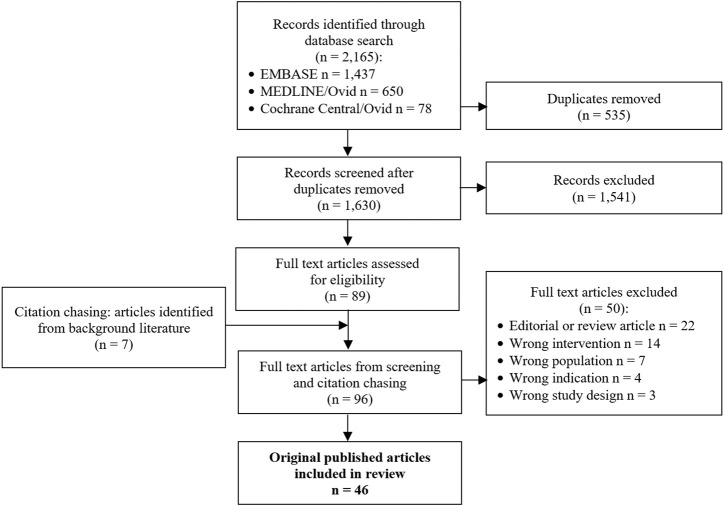
Preferred Reporting Items for Systematic Reviews and Meta-Analyses (PRISMA) flow diagram of study selection (Risk-based Eligibility Models Review, Europe, 2025).

Article selection involved independent screening of titles and abstracts by two reviewers (VEM, MA) against pre-defined criteria. Figure 1 shows the PRISMA-compliant selection process. A data extraction form was developed in Microsoft Excel before full-text review by the same two reviewers. RAYYAN.AI facilitated screening and inclusion.

Full article review and data extraction was done by two reviewers (VEM, MA). For each included study, we extracted (Supplementary Material S4): study description (e.g., title, author, publication year, population, design), model description (e.g., name, prediction outcome, type, time horizon), risk variables (e.g., age, smoking history, cancer history, lifestyle, comorbidities), performance description (e.g., AUC, C-statistic, calibration, sensitivity, specificity, risk thresholds), and outcome description (e.g., cases detected, LC-specific mortality reduction, QALY gained, ICER). To address the high heterogeneity of included risk models, tobacco exposure variables were grouped into standardized umbrella categories: ‘smoking duration’ encompassed any metric measuring the length of the smoking habit (e.g., total years smoked, categorized year-ranges), while ‘smoking intensity’ covered metrics quantifying the volume of tobacco consumed (e.g., average cigarettes per day, cumulative pack-years).

Discrepancies were resolved through discussion, with a third reviewer arbitrating (KTH, VR or TS). VEM contacted authors for desired but unreported data. Data underwent narrative synthesis, guided by PICO criteria, without limitations on sample size or model type. Potential study groupings were considered post-extraction.

## Results

Following the PRISMA framework (Figure 1), searches of EMBASE (n = 1,437), MEDLINE/Ovid (n = 650), and Cochrane Central/Ovid (n = 78) yielded 2,165 records. After 535 duplicates, 1,630 records were screened, resulting in 1,541 exclusions. Eighty-nine full-text articles were assessed, supplemented by 7 from citation chasing, for a total of 96. Of these, 50 full-text articles were excluded (editorial/review n = 22, wrong intervention n = 14, wrong population n = 7, wrong indication n = 4, wrong design n = 3). Ultimately, 46 articles (2003–2025) were included, comprising 35% observational, 7% descriptive, 17% modelling/simulation, 28% evaluation/validation, and 13% observational pilot designs (Supplementary Materials S5, S6).

### Existing risk-prediction models

**FIGURE 2:**
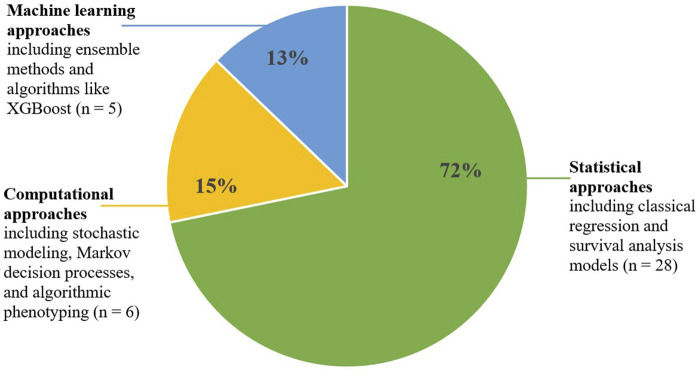
Risk-prediction models used for lung cancer screening by type of model approach (Risk-based Eligibility Models Review, Europe, 2025).

A total of 39 distinct risk-prediction models were identified across the 46 original published articles that met the inclusion criteria for this review (Table 1, Supplementary Table 2, Supplementary Materials S5, S7, S8). The predominant methodological approach employed in these models (72%) was statistical, encompassing classical regression techniques and survival analysis models. A further 15% of the identified models were categorized as computational approaches, including stochastic modelling, Markov decision processes, and algorithmic phenotyping. The remaining 13% of the assessed risk-prediction models utilized machine learning methodologies, incorporating ensemble methods and algorithms such as XGBoost (Figure 2, Supplementary Material S8).

**TABLE 1:** List of identified risk-prediction models (n = 39) (Risk-based Eligibility Models Review, Europe, 2025).

Name of LC risk-prediction model in assessed studies	Mention frequency (count)	Mention frequency (%)	Model type described in assessed studies	Prediction perspective	Prediction horizon (years)	Risk-variables (n)	References
PLCOm2012 model	23	50.0%	Logistic-regression model	Incidence	1 to 6	11	[13, 20–41]
Bach model	11	23.9%	Cox proportional hazards model; recursive estimation for projections beyond 1 year	Incidence and mortality	1 to 10	6	[13, 14, 21–23, 27, 29, 32, 34, 35, 42]
LCRAT (lung cancer incidence model)	7	15.2%	Cox proportional hazards model with non-parametric baseline hazards in prospective cohort	Incidence	5 to 6	11	[13, 21–23, 38, 43]
Liverpool lung project (LLP) model	7	15.2%	Logistic-regression model for relative risks; adjustment of intercept to match liverpool age-, sex, smoking-status incidence rates	Incidence	1 to 5	6–7	[13, 22, 23, 27, 32, 44, 45]
LCDRAT (lung cancer death risk assessment tool)	6	13.0%	Cox proportional hazards model with non-parametric baseline hazards in prospective cohort	Mortality	5 to 6	6–11	[13, 14, 21, 22, 43, 46]
Liverpool lung project (LLP) model v2	6	13.0%	Logistic-regression model for relative risks; adjustment of intercept to match liverpool age-, sex, smoking-status incidence rates	Incidence	5 to 6	8	[21, 24, 35, 37, 40, 47]
Liverpool lung project (LLP) model v3	5	10.9%	Logistic-regression model for relative risks; adjustment of intercept to match liverpool age-, sex, smoking-status incidence rates	Incidence	5	8	[21, 22, 35, 36, 47]
PLCOm2012 model simplified version	5	10.9%	Logistic-regression model	Incidence	6	6	[14, 32, 46, 48, 49]
Pittsburgh Predictor	4	8.7%	4-Factor logistic regression model	Incidence	6 to 8.7	4	[13, 22, 34, 35]
PLCOall2014	4	8.7%	Logistic-regression model	Incidence	6	12–13	[22, 33, 35, 36]
Hoggart model	2	4.3%	Weibull logistic-regression model in prospective cohort with stratification by status, age initiated smoking and quit years	Incidence	1 to 6	5	[13, 22]
HUNT model	2	4.3%	Multivariable cox regression model with non-linear transformations	Incidence	1 to 5	5–8	[21, 50]
Liverpool lung project (LLPi) incidence model	2	4.3%	Logistic-regression model for relative risks; adjustment of intercept to match liverpool age-, sex, smoking-status incidence rates	Incidence	5 to 8.7	7	[13, 22]
Lung-cancer death risk measure	2	4.3%	Multivariable regression model	Mortality	5	16	[13, 51]
OWL (optimized early warning model for LC risk) model	2	4.3%	XGBoost machine learning algorithm (ensemble)	Incidence	5 to 8	16–19	[21, 36]
PLCOm2012noRace model	2	4.3%	Logistic-regression model	Incidence	6	11	[52, 53]
Spitz 2007 lung cancer risk measure	2	4.3%	Logistic recursively cycling model for relative risks; attributable risk method applied to SEER incidence and mortality rates to obtain baseline rate	Incidence	1	14	[13, 22]
University College London Death (UCLD) model	2	4.3%	Machine learning model (ensemble)	Mortality	5	3	[21, 54]
University College London Incidence (UCLI) model	2	4.3%	Machine learning model (ensemble)	Incidence	5	3	[21, 54]
CanPredict (lung model), 10 years	1	2.2%	Cox proportional-hazards model	Incidence	10	15	[35]
CanPredict (lung model), 5 years	1	2.2%	Cox proportional-hazards model	Incidence	5	15	[35]
CanPredict (lung model), 6 years	1	2.2%	Cox proportional-hazards model	Incidence	6	15	[35]
Computable phenotype (CP) algorithms incl. EHR and NLP data	1	2.2%	Computable phenotype algorithm	Incidence	1	10	[55]
COSMOS model	1	2.2%	Cox proportional-hazards model	Incidence	1	8	[56]
Knoke model	1	2.2%	Two-parameter Poisson regression model	Mortality	6 to 10	5	[32]
LCRAT + CT	1	2.2%	Cox proportional hazards model	Next-screen risk	5	10	[57]
Liverpool lung project (LLP) model simplified version	1	2.2%	Logistic-regression model for relative risks; adjustment of intercept to match liverpool age-, sex, smoking-status incidence rates	Incidence	5	6	[32]
Lung cancer screening decision (ENGAGE) tool	1	2.2%	Partially observable markov decision process (POMDP)	Incidence	Up to 100 (markov)	5	[46, 58]
LungFlag model	1	2.2%	Machine learning algorithm	Incidence	1	5	[59]
Medial EarlySign (MES) machine learning model	1	2.2%	XGBoost (extreme gradient boosting)	Incidence	1	7	[60]
Pan-canadian early detection of lung cancer (PanCan) model	1	2.2%	Logistic-regression model	Incidence	6	7	[61]
PLCO2012 result model	1	2.2%	Logistic-regression model	Incidence	1 to 6	12	[62]
PLCOm2012 Race3L	1	2.2%	Logistic-regression model	Incidence	6	12	[63]
PLCOm2012bu model	1	2.2%	Logistic-regression model	Incidence	6	11	[62]
Polynomial model	1	2.2%	Logistic-regression model	Incidence	NA	8	[57]
Safety net Hospitals (SNH) model	1	2.2%	Logistic-regression model	Incidence	6	5	[26]
Two-stage clonal expansion (TSCE) CPS LC death model	1	2.2%	Stochastic representation of the cell events	Mortality	1	6	[32]
Two-stage clonal expansion (TSCE) LC incidence model	1	2.2%	Stochastic representation of the cell events	Incidence	1	6	[32]
Two-stage clonal expansion (TSCE) NHS/HPFS LC death model	1	2.2%	Stochastic representation of the cell events	Mortality	1	6	[32]

The most frequently used or assessed risk prediction model (50%; 23/46) was the Tammemägi PLCOm2012 model, which incorporates 11 risk variables [20]. The Bach Model was the second most described and validated model appearing in 24% (11/46) of the included articles [42]. Further models mentioned in 4% and more of the studies were: the Lung Cancer Incidence and Death Risk Assessment Tool (LCRAT, LCDRAT) [43], the Liverpool Lung Project (LLP) models [13, 45, 47], further PLCO models like the simplified version [32], the noRace model [52], and the PLCOall2014 model [33], the Pittsburgh Predictor [34], the Hoggart Model [13], the HUNT model [50], the Lung-cancer Death Risk Measure [51], the OWL (Optimized Early Warning Model for LC Risk) model [36], the Spitz model [13], the University College London Death and Incidence models (UCLD and UCLI) [21]. All identified models are presented in Supplementary Material S7, additional overviews in Table 1, Supplementary Table 2 and outcomes in Supplementary Table 3.

### Identification of eligible populations utilizing complex risk prediction

**FIGURE 3:**
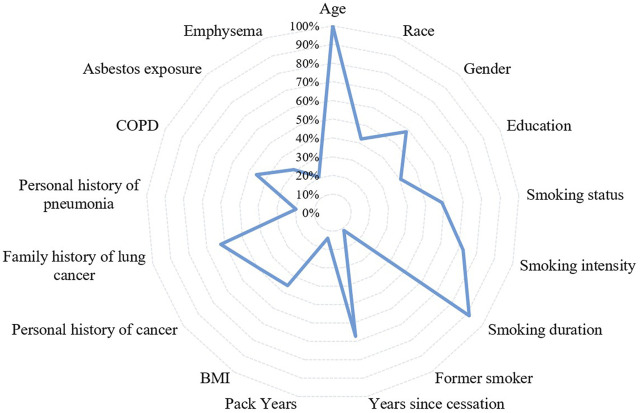
Most frequently used lung cancer risk variables in assessed models (≥10% mentions) (Risk-based Eligibility Models Review, Europe, 2025).

The literature indicates that risk-prediction models offer a refined approach to identifying populations eligible for LCS compared to solely age cut-offs and tobacco exposure criteria. These models incorporate diverse risk variables, allowing for more accurate stratification of individuals at high risk for developing LC. The identified risk prediction models utilized varying combinations of risk factors to determine screening eligibility. In assessed models the top five frequently utilized variables were: age (100%), smoking duration (91.4%), smoking intensity (72.4%), years since cessation (67.2%), family history of lung cancer (62.1%). These results demonstrate that model precision stems from integrating essential age and smoking metrics with secondary clinical and demographic variables, rather than using them in isolation. Supplementary Table 2 details the risk models, including the variables used and relevant study characteristics. Supplementary Table 3 presents the outcomes for each model and assessed study. Supplementary Material S7 describes which variables are applied how frequently in each model, and a relationship further visualized in Figure 3.

The diversity of risk-variables suggests tailored strategies for identifying high-risk populations. Studies show these models can significantly improve eligible individual identification. For example, the PLCOm2012 model (11 risk-variables, ≥1.51% threshold) identified more lung cancer screening candidates in the Yorkshire Lung Screening Trial (ever smokers aged 55–80 years) and resulted in higher screen-detected cancer rates than the LLPv2 model (>5% threshold) or USPSTF 2013 criteria [40]. Similarly, Roe et al. highlighted that traditional criteria miss a significant proportion of future lung cancer cases in a high-risk population of smokers (median age 57.6, BMI 24.75, packyears 33.8, cigarettes per day 20), supporting the use of models like the HUNT model [50]. Bach et al.'s work suggests that risk models can better target high-risk individuals within standard screening cohorts [42]. A study by Bhardwaj et al. assessed 11 lung cancer (LC) risk models using AUC in the 17-year ESTHER prospective cohort (German screening-age adults), comparing them to LDCT trial criteria. The Bach, LCRAT, and LCDRAT models demonstrated superior LC incidence risk prediction among ESTHER ever-smokers, achieving AUCs of 0.782–0.787 (6 years), 0.770–0.774 (11 years), and 0.765–0.771 (17 years). Significantly, these models detected 11.8%–17.6% more LC cases within 6 years at positivity rates similar to LDCT trial criteria. This highlights their potential to improve high-risk identification and boost LC screening’s mortality reduction efficacy [22].

Hüsing et al. offer specific European guidance for lung cancer (LC) risk prediction, suggesting a pragmatic LDCT LC-screening approach for 50–75 year olds with smoking history and a ≥1.6% 5-year LC risk (e.g., via PLCOm2012). Their analysis of German survey data (2008–2013) estimates this strategy would involve 3.0 million individuals and account for 40% of new LC diagnoses. The authors underscore the critical role of customized simulation models for specific populations (e.g., Germany) to rigorously evaluate screening benefits based on absolute risk thresholds. They also recommend exploring the inclusion of older individuals and those with comorbidities (e.g., COPD), and advocate for sex-specific simulation studies to optimize screening efficiency, particularly given potential greater mortality reduction in women from CT screening [23].

### Additional impact of risk-prediction tools

Ten Haaf et al. retrospectively analysed NLST and PLCO data, evaluating nine LC risk prediction models for calibration, discrimination, and utility in predicting 6-year LC incidence and mortality [32]. All models showed satisfactory calibration, but discriminative abilities varied (AUC 0.61–0.80) due to population risk factor heterogeneity. Importantly, all nine models demonstrated superior sensitivity and specificity compared to NLST eligibility criteria. PLCOm2012, Bach, and Two-Stage Clonal Expansion incidence models exhibited the strongest overall performance (AUC 0.69–0.80, 0.68–0.79, and 0.67–0.79, respectively), suggesting these models can refine LCS selection.

Similarly, Feng et al. validated ten risk prediction models in European cohorts (Lung Cancer Cohort Consortium, n = 240,137 participants aged 45–80 with a smoking history), assessing calibration and discrimination [21]. Most models showed reasonable calibration, though Liverpool Lung Project (LLP) version 2 consistently overpredicted risk. Discrimination was similar across models, with some AUC variability (Supplementary Table 3). Several models identified comparable or higher numbers of future LC cases versus categorical criteria like USPSTF-2021 [21].

Model discriminative power - the ability to correctly distinguish individuals who will develop lung cancer from those who will not, typically measured by the Area Under the Curve (AUC) - varied significantly across the assessed studies (AUCs ranging from 0.61 to 0.89) [21, 32]. This wide variability represents a critical implementation issue. Models demonstrating lower discriminative power, often due to their reliance on broad categorical ranges rather than continuous data for tobacco exposure variables like smoking duration and intensity, fail to accurately separate high-risk from low-risk individuals [21]. Consequently, utilizing a model with suboptimal discriminative power can severely compromise screening efficiency by simultaneously increasing false-positive referrals and missing true cancer cases.

Beyond performance evaluation, other studies provide insights into real-world implementation and cost-effectiveness. Roe et al. highlighted risk models’ utility for individual ranking and cost-effective screening thresholds, favouring the HUNT Lung Cancer Model [50]. Kats et al. showed risk models outperform NLST criteria in real-world settings [38]. Tammemägi et al. demonstrated high LC detection (2.4%) and early-stage diagnosis (79.2%), with strong follow-up adherence (>85%) in a universal healthcare setting [52]. However, Jungblut et al. noted challenges in reaching all at-risk groups, observing an overrepresentation of highly educated participants (82%) [39]. Tammemägi et al. explored adjusting screening intervals based on initial risk and subsequent negative screens [62], while Bartlett et al. examined different risk thresholds, finding LLPv2 identified more scan-eligible participants than PLCOm2012 [24].

Cost-effectiveness analyses of lung cancer screening consistently highlight advantages of risk-based eligibility models over traditional pack-year criteria. While some studies suggest comparable cost-effectiveness in specific contexts [48], comprehensive comparative modelling often indicates that risk-based approaches offer a more favourable balance of outcomes [46]. For example, a simulation by Tomonaga et al. [49] found their risk-based “RISK11” strategy (biennial screening for 55–80 year olds with 1.6% PLCOm2012 LC risk) to be among the most feasible and cost-effective, significantly reducing both costs and CT scans while maintaining comparable Quality-Adjusted Life Years (QALYs). RISK11 also demonstrated lower average and incremental cost-effectiveness ratios (ACER and ICER) compared to other approaches [49]. Similarly, Roseleur et al. identified PLCOm2012 risk-model scenarios as cost-effective in Australia, achieving substantial mortality reduction and QALYs with fewer LDCT screens than standard recommendations [48]. Further supporting this, Toumazis et al. found risk model-based screening strategies more cost-effective than USPSTF recommendations, exclusively occupying the cost-effectiveness efficiency frontier in their comparative analysis (annual LDCT screening, aged 50–55 years, 6-year risk thresholds 0.5%–2.2% PLCOm2012 model) [46]. Sensitivity analyses consistently confirmed the robustness of these findings across various studies, despite key parameters like LDCT cost, specificity, and screening disutility influencing overall cost-effectiveness [46, 48, 49, 64]. Despite these sensitivities, risk model-based strategies remained robustly more cost-effective than other approaches under varying modelling assumptions.

### Comparison of risk models

When comparing models developed in different regions, several large prospective studies demonstrated that models developed and trained with data from other geographical region, often perform as well as, or better than, European-developed models when applied to European cohorts. In a comprehensive validation of ten models across nine European countries (n = 240,137), Feng et al. found that models trained on US-datasets (such as PLCOm2012, LCDRAT, LCRAT, and Bach) achieved similar or superior discrimination (AUCs ranging from 0.68 to 0.83) compared to models trained on European datasets like the Liverpool Lung Project (LLP) versions 2 and 3 (AUCs 0.64–0.78) [21]. Similarly, Bhardwaj et al. evaluated 11 models in a German prospective cohort over 17 years and found that the US-developed Bach, LCRAT, and LCDRAT models provided the most accurate risk prediction, outperforming European models like LLP and the Hoggart model [22]. These findings suggest that robust statistical design and the comprehensiveness of the included risk variables - such as utilizing continuous data for smoking intensity rather than broad categories - are the critical determinants of a model’s utility, rendering these well-calibrated US models highly applicable to European screening programs.

## Discussion

### Main findings

This systematic review synthesized the evidence from 46 studies, identifying 39 distinct lung cancer risk prediction models. The predominant methodological approaches observed were statistical modelling, followed by computational and machine learning techniques, with the PLCOm2012 model being the most frequently investigated.

The collective findings from this review and the broader literature strongly support the potential of risk-based models to refine lung cancer screening strategies. While age and smoking metrics remain the most frequently used risk variables, incorporating a diverse array of supplementary risk factors (such as family history, BMI, and comorbidities) – rather than relying exclusively on traditional age and smoking history cut-offs - allows these models to offer a more precise approach to identifying individuals at high risk. Evidence suggests that these models optimize efficiency by identifying higher-yield screening populations [40]. Instead of screening more people, well-calibrated models substitute lower-risk individuals for high-risk cases missed by standard criteria [50], thereby improving detection rates without increasing over-screening [22]. However, performance metrics, with AUC values ranging from 0.61 to 0.81, indicate to assess calibration and discrimination ability based on local population data intended for LCS [32]. Furthermore, several studies highlight the practical implications of utilizing risk prediction models and algorithms directly within EHR systems. Kats et al. [38] demonstrated their utility by outperforming NLST criteria, while Yang et al. [55] confirmed the effectiveness of EHR-based computable phenotypes – integrating both structured data and unstructured clinical notes – to accurately automate the identification of LCS-eligible individuals in real-world clinical settings. Feng et al. [21] suggest that validated models can enhance screening efficiency in European cohorts, albeit with a need for local calibration. The cost-effectiveness analyses by Roseleur et al. [48], Tomonaga et al. [49] and Toumazis et al. [46] indicate that risk-based screening can be a resource-efficient approach, potentially even superior to pack-year-based strategies. Moreover, Tammemägi et al. [62] propose that risk scores can inform personalized screening intervals and guide enrollment in clinical trials. Hüsing et al.'s work provides specific recommendations for implementing risk prediction in a European context, emphasizing the need for tailored simulation modelling [23].

However, the heterogeneity in the included risk factors and the variability in model performance across different populations underscore the complexity of this field. While promising, the optimal implementation of risk prediction models requires careful consideration of model calibration to specific populations, as highlighted by Feng et al. [21]. Further research is warranted to validate these models in diverse real-world settings, to explore the integration of novel biomarkers and risk factors, and to assess the long-term impact on lung cancer incidence and mortality.

### Limitations

Given that this review is intended to inform public health and health policy discussions on the inclusion of risk-eligibility models in LCS approaches to better identify the high-risk population within Europe, studies based on East-Asian populations were intentionally excluded. This exclusion was a deliberate methodological choice due to documented differences in LC aetiology, genetics, and progression, which limit the direct comparability of such risk models. Consequently, a limitation of our review is the lack of generalizability of our findings to that specific region. Another difficulty is that the inclusion of 39 distinct risk prediction models employing varied methodologies (statistical, computational, and machine learning) and diverse risk factor sets makes direct, comprehensive comparison of performance challenging. Third, the evidence is dominated by statistical approaches (72%), with fewer studies utilizing and validating newer computational (15%) or machine learning (13%) methods, which limits the conclusions regarding the comparative utility of these emerging technologies. And finally, the use of narrative synthesis, as opposed to a quantitative meta-analysis, was necessary due to the heterogeneity of the extracted data, which precludes generating combined statistical estimates of model performance.

### Conclusion

This systematic review underscores the significant potential of lung cancer risk prediction models to improve the efficiency and effectiveness of screening programs. The identified models offer a more sophisticated approach to identifying high-risk individuals compared to traditional criteria, with evidence suggesting benefits in terms of detection rates, cost-effectiveness, and personalized screening strategies. The PLCOm2012 model stands out as the most frequently studied, indicating its established role in the field.

The findings from this review, coupled with the evidence from other studies, advocate for a greater consideration and implementation of risk-based approaches in LCS. However, the heterogeneity of existing models and the need for population-specific validation and calibration remain critical challenges. Future research should focus on refining and validating these models in diverse settings, exploring their integration into clinical workflows, and assessing their impact on patient outcomes. Ultimately, the judicious application of risk prediction models holds promise for optimizing LCS and potentially reducing the burden of this disease.
