# Supplementary material for: Use and impact of risk-based eligibility models in low-dose computed tomography lung cancer screening: a systematic review
Source: Public Health Rev. 2026 Jul 20;47:1609133. doi: 10.3389/phrs.2026.1609133 (PMC13430644; doi:10.3389/phrs.2026.1609133)
Supplement: Supplementary file 1 [file Table1.docx]

**Supplementary Table 1: Overview of the assessed 39 risk-prediction models described along identified studies (n = 39) (Risk-based Eligibility Models Review, Europe, 2025).**

| **Model Description** | | | | | **Study Description** | | | |
| --- | --- | --- | --- | --- | --- | --- | --- | --- |
| **Name of LC Risk-Prediction Model in assessed studies** | **Model type described in assessed studies** | **Prediction perspective** | **Prediction time horizon (years)** | **Number of included risk-variables** | **Study first author, year published** | **Study population described in study** | **Age** | **Described study type** |
| Bach model | Cox proportional hazards model; recursive estimation for projections beyond 1 year | incidence | 5 | 6:  Age; Gender; Smoking duration; Smoking intensity; Years since cessation; Asbestos exposure | Feng X et al. 2024 [21] | 240,137 Current/former smokers from 9 European countries (Finland, France, Denmark, Germany, Italy, Spain, Sweden, Netherlands, Norway) | 45-80 | Prospective cohort consortium analysis |
|  |  | incidence | 1 |  | Ten Haaf K et al. 2017 [32] | 134,124 NLST & PLCO Ever-Smokers | 57-69 | Retrospective validation study |
|  |  | incidence | 5 |  | Ostrowski M et al. 2021 [27] | 6,631 Ever-Smokers, ≥30 pack-years smoking history | 50-79 | comparative study with cohort data MOLTEST BIS programme |
|  |  | incidence | 1 |  | Bhardwaj M et al. 2022 [22] | 9,407 Population from ESTHER cohort study | 50-75 | Comparative evaluation study |
|  |  | incidence | 1 |  | Meza R et al. 2021 [14] | 1,000,000 1950 and 1960 US birth cohort | 45-90 | Comparative simulation modeling study based on four microsimulation models (CISNET) |
|  |  | incidence | 6 |  | Cleven K et al. 2021 [29] | 3,953 Ever-Smokers, FDNY-WTC exposed rescue and recovery workers (firefighters/EMS) | 50-80 | Retrospective analysis |
|  |  | incidence | 5 |  | Hüsing A et al. 2020 [23] | 16 Mio. projected from 14,834 Ever-Smokers of German population study GEDA 2008-2013 and 20.700 Ever-Smokers from German EPIC cohort (EPIC-D) | 50-79 | Retrospective analysis |
|  |  | incidence | 10 |  | Katki H et al. 2018 [13] | 409,726 Ever-Smokers from National Institutes of Health–AARP Diet and Health Study (NIH AARP) and Ever-Smokers from CPS-II (Cancer Prevention Study II) Nutrition Survey cohort | 50-80 | Comparison and Validation Study |
|  |  | mortality | 1 |  | Wilson D et al. 2015 [34] | 57,108 Current and former smokers (PLuSS and NLST | 50-79 | Original quantitative evaluation of risk prediction models |
|  |  | incidence | 1 |  | Bach P et al. 2003 [42] | 18,314 Heavy smoker and asbestos cohort | 44-75 | modeling study based on CARET cohort study |
|  |  | mortality | 10 |  | Liao W et al. 2023 [35] | 19,670,000 Asymptomatic adults from English primary care databases (QResearch & CPRD) | 25-84 | Retrospective population-based cohort study |
| CanPredict (lung model), 10 years | Cox proportional hazards model | incidence | 10 | 15:  Age; Gender; Race; Smoking status; Smoking intensity; Asbestos exposure; Personal history of cancer; Family history of lung cancer; Personal history of pneumonia; BMI; COPD; Asthma; Venous thromboembolism; Alcohol; Socio economic status (Townsend score) | Liao W et al. 2023 [35] | 19,670,000 Asymptomatic adults from English primary care databases (QResearch & CPRD) | 25-84 | Retrospective population-based cohort study |
| CanPredict (lung model), 5 years |  |  | 5 |  | Liao W et al. 2023 [35] | 19,670,000 Asymptomatic adults from English primary care databases (QResearch & CPRD) | 25-84 | Retrospective population-based cohort study |
| CanPredict (lung model), 6 years |  |  | 6 |  | Liao W et al. 2023 [35] | 19,670,000 Asymptomatic adults from English primary care databases (QResearch & CPRD) | 25-84 | Retrospective population-based cohort study |
| Computable phenotype (CP) algorithms for LCS eligibility, Rule-based algorithm combining structured EHR data and NLP-processed unstructured data | Computable phenotype algorithm | incidence | 1 | 10:  Age; Smoking status; Former smoker; Current smoker; Smoking duration; Smoking intensity; Years since cessation; Pack Years; Personal history of cancer; Family history of lung cancer | Yang S et al. 2025 [55] | 5,778 Individuals from University of Florida Health Integrated Data Repository (2012–2022 LDCT recipients) | 50-80 | Original quantitative evaluation study |
| COSMOS model | Cox proportional hazards model | incidence | 1 | 8:  Age; Gender; Smoking status; Smoking duration; Smoking intensity; Years since cessation; Asbestos exposure; COPD | Maisonneuve P et al. 2023 [56] | 5,203 Asymptomatic individuals aged ≥50, heavy smokers (≥20 pack-years) | 50-84 | Prospective cohort study (based on the COSMOS trial) |
| Hoggart Model | Weibull logistic-regression model in prospective cohort with stratification by status, age initiated smoking and quit years | incidence | 6 | 5:  Age; Smoking status; Smoking duration; Smoking intensity; Years since cessation | Bhardwaj M et al. 2022 [22] | 9,408 Population from ESTHER cohort study | 50-75 | Comparative evaluation study |
|  |  |  | 1 to 5 |  | Katki H et al. 2018 [13] | 409,726 Ever-Smokers from National Institutes of Health–AARP Diet and Health Study (NIH AARP) and Ever-Smokers from CPS-II (Cancer Prevention Study II) Nutrition Survey cohort | 50-80 | Comparison and Validation Study |
| HUNT model (Norwegian Nord-Trøndelag Health Study model) | Multivariable Cox regression model with non-linear transformations | incidence | 6 | 8:  Age; Gender; Smoking intensity; Years since cessation; Pack Years; Smoke exposure hours per day; Daily cough; BMI | Feng X et al. 2024 [21] | 240,137 Current/former smokers from 9 European countries (Finland, France, Denmark, Germany, Italy, Spain, Sweden, Netherlands, Norway) | 45-80 | Prospective cohort consortium analysis |
|  |  |  | 10 |  | Roe O D et al. 2019 [50] | 4,051 DLST cohort participants | 50-70 | Retrospective analysis of DLCST cohort data |
| Knoke model | Two-parameter Poisson regression model | mortality | 1 | 5:  Age; Smoking status; Smoking duration; Smoking intensity; Years since cessation | Ten Haaf K et al. 2017 [32] | 134,124 NLST & PLCO Ever-Smokers | 57-69 | Retrospective validation study |
| LCDRAT (Lung Cancer Death Risk Assessment Tool) | Cox proportional hazards model with non-parametric baseline hazards in prospective cohort | mortality | 5 | 11:  Age; Gender; Race; Smoking duration; Smoking intensity; Years since cessation; Pack Years; Family history of lung cancer; Emphysema; Education; BMI  6:  Age; Gender; Smoking status; Smoking duration; Smoking intensity; Years since cessation | Feng X et al. 2024 [21] | 240,137 Current/former smokers from 9 European countries (Finland, France, Denmark, Germany, Italy, Spain, Sweden, Netherlands, Norway) | 45-80 | Prospective cohort consortium analysis |
|  |  |  | 5 |  | Katki H et al. 2016 [43] | 132,160 PLCO & NLST Ever-Smokers | 55-74 | Development and Validitation Study |
|  |  |  | 6 |  | Toumazis I et al. 2023 [46] | 1,000,000 1960 US birth cohort | 45-90 | Comparative modeling analysis based on four microsimulation models (CISNET) |
|  |  |  | 5 |  | Bhardwaj M et al. 2022 [22] | 9,411 Population from ESTHER cohort study | 50-75 | Comparative evaluation study |
|  |  |  | 5 |  | Meza R et al. 2021 [14] | 1,000,000 1950 and 1960 US birth cohort | 45-90 | Comparative simulation modeling study based on four microsimulation models (CISNET) |
|  |  |  | 5 |  | Katki H et al. 2018 [13] | 409,726 Ever-Smokers from National Institutes of Health–AARP Diet and Health Study (NIH AARP) and Ever-Smokers from CPS-II (Cancer Prevention Study II) Nutrition Survey cohort | 50-80 | Comparison and Validation Study |
| LCRAT (Lung Cancer Incidence Model) | Cox proportional hazards model with non-parametric baseline hazards in prospective cohort | incidence | 5 | 11:  Age; Gender; Race; Smoking status; Smoking duration; Smoking intensity; Years since cessation; Family history of lung cancer; Emphysema; Education; BMI | Feng X et al. 2024 [21] | 240,137 Current/former smokers from 9 European countries (Finland, France, Denmark, Germany, Italy, Spain, Sweden, Netherlands, Norway) | 45-80 | Prospective cohort consortium analysis |
|  |  |  | 5 |  | Katki H et al. 2016 [43] | 132,160 PLCO & NLST Ever-Smokers | 55-74 | Development and Validitation Study |
|  |  |  | 5 |  | Bhardwaj M et al. 2022 [22] | 9,410 Population from ESTHER cohort study | 50-75 | Comparative evaluation study |
|  |  |  | 6 |  | Kats et al. 2021 [38] | 37,327 Ever-Smokers | 55-74 | Retrospective cohort validation study based on EHR |
|  |  |  | 5 |  | Hüsing A et al. 2020 [23] | 16 Mio. projected from 14,834 Ever-Smokers of German population study GEDA 2008-2013 and 20.700 Ever-Smokers from German EPIC cohort (EPIC-D) | 50-79 | Retrospective analysis |
|  |  |  | 5 |  | Katki H et al. 2018 [13] | 409,726 Ever-Smokers from National Institutes of Health–AARP Diet and Health Study (NIH AARP) and Ever-Smokers from CPS-II (Cancer Prevention Study II) Nutrition Survey cohort | 50-80 | Comparison and Validation Study |
|  |  |  | 5 |  | Liao W et al. 2023 [35] | 19,670,000 Asymptomatic adults from English primary care databases (QResearch & CPRD) | 25-84 | Retrospective population-based cohort study |
| LCRAT+CT | Cox proportional hazards model | next-screen risk | NA | 10:  Age; Race; Smoking duration; Years since cessation; Pack Years; Family history of lung cancer; Emphysema; Education; BMI; COPD | Maldonado S et al. 2021 [57] | 4,052 Ever-Smokers | 50-69 | Validation Study |
| Liverpool Lung Project (LLP) model | Logistic-regression model for relative risks; adjustment of intercept to match Liverpool age-, sex, smoking-status incidence rates | incidence | 5 | 7:  Age; Gender; Smoking duration; Asbestos exposure; Personal history of cancer; Family history of lung cancer; Personal history of pneumonia | Ten Haaf K et al. 2017 [32] | 134,124 NLST & PLCO Ever-Smokers | 57-69 | Retrospective validation study |
|  |  |  | 5 |  | Ostrowski M et al. 2021 [27] | 6,631 Ever-Smokers, ≥30 pack-years smoking history | 50-79 | comparative study with cohort data MOLTEST BIS programme |
|  |  |  | 5 |  | Bhardwaj M et al. 2022 [22] | 9,409 Population from ESTHER cohort study | 50-75 | Comparative evaluation study |
|  |  |  | 5 |  | Hüsing A et al. 2020 [23] | 16 Mio. projected from 14,834 Ever-Smokers of German population study GEDA 2008-2013 and 20.700 Ever-Smokers from German EPIC cohort (EPIC-D) | 50-79 | Retrospective analysis |
|  |  |  | 5 |  | Katki H et al. 2018 [13] | 409,726 Ever-Smokers from National Institutes of Health–AARP Diet and Health Study (NIH AARP) and Ever-Smokers from CPS-II (Cancer Prevention Study II) Nutrition Survey cohort | 50-80 | Comparison and Validation Study |
|  |  |  | 5 |  | Raji O et al. 2012 [44] | 2,922 EUELC (European), Harvard (North American), and LLPC (UK) studies | 40-79 | Case-control & prospective cohort |
|  |  |  | 1 |  | Cassidy A et al. 2007 [45] | 1,736 Residents of the Liverpool area, incident cases of histologically or cytologically confirmed lung cancer were between 20 and 80 years of age | 57-75 | Case-control study |
| Liverpool Lung Project (LLP) model simplified version | Logistic-regression model for relative risks; adjustment of intercept to match Liverpool age-, sex, smoking-status incidence rates | incidence | 5 | 6:  Age; Gender; Smoking status; Smoking duration; Smoking intensity; Years since cessation | Ten Haaf K et al. 2017 [32] | 134,124 NLST & PLCO Ever-Smokers | 57-69 | Retrospective validation study |
| Liverpool Lung Project (LLP) model version 2 | Logistic-regression model for relative risks; adjustment of intercept to match Liverpool age-, sex, smoking-status incidence rates | incidence | 5 | 8:  Age; Gender; Smoking duration; Asbestos exposure; Personal history of cancer; Family history of lung cancer; Personal history of pneumonia; COPD | Feng X et al. 2024 [21] | 240,137 Current/former smokers from 9 European countries (Finland, France, Denmark, Germany, Italy, Spain, Sweden, Netherlands, Norway) | 45-80 | Prospective cohort consortium analysis |
|  |  |  | 6 |  | Lebrett M et al. 2020 [37] | 1,429 Ever-Smokers Manchester Lung Health Check (LHC) pilot | 55-74 | Comparison Study |
|  |  |  | 5 |  | Gabe R et al. 2024 [40] | 7,826 Ever-Smokers in Yorkshire, UK | 55-80 | Prospective cohort study (randomized controlled trial with community-based LDCT-LCS) |
|  |  |  | 5 |  | Field J K et al. 2021 [47] | 75,958 Risk population | 50-79 | Case-control and prospective cohort designs |
|  |  |  | 5 |  | Bartlett E et al. 2020 [24] | 8,366 Ever-Smokers in West London, UK | 60-75 | Prospective observational pilot study |
|  |  |  | 5 |  | Liao W et al. 2023 [35] | 19,670,000 Asymptomatic adults from English primary care databases (QResearch & CPRD) | 25-84 | Retrospective population-based cohort study |
| Liverpool Lung Project (LLP) model version 3 | Logistic-regression model for relative risks; adjustment of intercept to match Liverpool age-, sex, smoking-status incidence rates | incidence | 5 | 8:  Age; Gender; Smoking duration; Asbestos exposure; Personal history of cancer; Family history of lung cancer; Personal history of pneumonia; COPD | Feng X et al. 2024 [21] | 240,137 Current/former smokers from 9 European countries (Finland, France, Denmark, Germany, Italy, Spain, Sweden, Netherlands, Norway) | 45-80 | Prospective cohort consortium analysis |
|  |  |  |  |  | Pan Z et al. 2023 [36] | 323,344 General population (UK Biobank, UKB) | 37-73 | Development and validation study |
|  |  |  |  |  | Bhardwaj M et al. 2022 [22] | 9,417 Population from ESTHER cohort study | 50-75 | Comparative evaluation study |
|  |  |  |  |  | Field J K et al. 2021 [47] | 75,958 Risk population | 50-79 | Case-control and prospective cohort designs |
|  |  |  |  |  | Liao W et al. 2023 [35] | 19,670,000 Asymptomatic adults from English primary care databases (QResearch & CPRD) | 25-84 | Retrospective population-based cohort study |
| Liverpool Lung Project (LLPi) incidence model | Logistic-regression model for relative risks; adjustment of intercept to match Liverpool age-, sex, smoking-status incidence rates | incidence | 5 | 7:  Age; Gender; Smoking duration; Personal history of cancer; Family history of lung cancer; Emphysema; COPD | Bhardwaj M et al. 2022 [22] | 9,416 Population from ESTHER cohort study | 50-75 | Comparative evaluation study |
|  |  |  | 8,7 |  | Katki H et al. 2018 [13] | 409,726 Ever-Smokers from National Institutes of Health–AARP Diet and Health Study (NIH AARP) and Ever-Smokers from CPS-II (Cancer Prevention Study II) Nutrition Survey cohort | 50-80 | Comparison and Validation Study |
| Lung cancer screening decision (ENGAGE) tool | Partially observable Markov decision process (POMDP) | incidence | up to 100 (Markov) | 5:  Age; Race; Smoking duration; Smoking intensity; Years since cessation | Toumazis I et al. 2020 [58] | Subpopulation of Ever-Smokers | 50-80 | Original quantitative evaluation study using partially observable Markov decision process (POMDP) |
| Lung-cancer Death Risk Measure | Multivariable regression model | mortality | 5 | 16:  Age; Gender; Race; Smoking status; Former smoker; Current smoker; Smoking duration; Smoking intensity; Years since cessation; Asbestos exposure; Dust exposure; Family history of lung cancer; Personal history of pneumonia; Emphysema; BMI; COPD | Katki H et al. 2018 [13] | 409,726 Ever-Smokers from National Institutes of Health–AARP Diet and Health Study (NIH AARP) and Ever-Smokers from CPS-II (Cancer Prevention Study II) Nutrition Survey cohort | 50-80 | Comparison and Validation Study |
|  |  | mortality | 5 |  | Kovalchik S et al. 2013 [51] | 53,158 LDCT Group & Radiography Group | 55-74 | Modeling Study |
| LungFlag model | Machine learning algorithm | incidence | 1 | 5+:  Age; Gender; Smoking status; Laboratory data; Clinical variables (blood exams) | Trujillo J C et al. 2025 [59] | 3,835,128 Individuals meeting USPSTF 2013 criteria in Spain (adults aged 55-80 with ≥30 pack-year smoking history) | 55-80 | Cost-effectiveness analysis using decision-tree + Markov model |
| Medial EarlySign (MES) machine learning model | XGBoost (Extreme Gradient Boosting) | incidence | 1 | 7+:  Age; Former smoker; Years since cessation; Daily cough; Education; Venous thromboembolism; Alcohol | Gould M et al. 2021 [60] | 196,102 NSCLC case patients | 45-90 | Retrospective cohort study, Kaiser Permanente Southern California (KPSC) |
| OWL (Optimized Early Warning Model for Lung Cancer Risk) model | XGBoost machine learning algorithm (ensemble) | incidence | 8 | 13:  Age; Gender; Smoking status; Smoking duration; Smoking intensity; Years since cessation; Age when start smoking; Pack Years; Family history of lung cancer; Education; BMI; COPD; Diabetes | Feng X et al. 2024 [21] | 240,137 Current/former smokers from 9 European countries (Finland, France, Denmark, Germany, Italy, Spain, Sweden, Netherlands, Norway) | 45-80 | Prospective cohort consortium analysis |
|  |  | incidence | 5 und 6 | 19:  Age; Gender; Smoking status; Smoking duration; Smoking intensity; Years since cessation; Age when start smoking; Pack Years; Asbestos exposure; Dust exposure; Smoke exposure hours per day; Family history of lung cancer; Personal history of pneumonia; Emphysema; Education; BMI; COPD; Diabetes; Alcohol | Pan Z et al. 2023 [36] | 323,344 General population (UK Biobank, UKB) | 37-73 | Development and validation study |
| Pan-Canadian Early Detection of Lung Cancer (PanCan) Model | Logistic-regression model | incidence | 6 | 7:  Age; Smoking duration; Pack Years; Family history of lung cancer; Education; BMI; COPD | Tammemägi M et al. 2017 [61] | 7,044 Ever-Smokers aged 50–75 years without lung cancer history, recruited across eight Canadian centers | 50-75 | Singe-arm, prospective study |
| Pittsburgh Predictor | 4-factor logistic regression model | incidence | 6 | 4:  Age; Smoking status; Smoking duration; Smoking intensity | Bhardwaj M et al. 2022 [22] | 9,414 Population from ESTHER cohort study | 50-75 | Comparative evaluation study |
|  |  | incidence | 8,7 |  | Katki H et al. 2018 [13] | 409,726 Ever-Smokers from National Institutes of Health–AARP Diet and Health Study (NIH AARP) and Ever-Emokers from CPS-II (Cancer Prevention Study II) Nutrition Survey cohort | 50-80 | Comparison and Validation Study |
|  |  | incidence | 6 |  | Wilson D et al. 2015 [34] | 57,108 Current and former smokers (PLuSS and NLST | 50-79 | Original quantitative evaluation of risk prediction models |
|  |  | incidence | 6 |  | Liao W et al. 2023 [35] | 19,670,000 Asymptomatic adults from English primary care databases (QResearch & CPRD) | 25-84 | Retrospective population-based cohort study |
| PLCO2012 result model | Logistic-regression model | incidence | 1-4 | 12:  Age; Race; Smoking status; Smoking duration; Smoking intensity; Years since cessation; Personal history of cancer; Family history of lung cancer; Education; BMI; COPD; Incl prev. PLCOm2012 scores and LungRADS results | Tammemägi M et al. 2019 [62] | 22,229 Ever-Smokers, NLST participants (LSS and ACRIN subsets) | 57-65 | Secondary analysis of NLST RCT data |
| PLCOall2014 | Logistic-regression model | incidence | 6 | 13:  Age; Race; Smoking status; Former smoker; Current smoker; Smoking duration; Smoking intensity; Years since cessation; Personal history of cancer; Family history of lung cancer; Education; BMI; COPD | Pan Z et al. 2023 [36] | 323,344 General population (UK Biobank, UKB) | 37-73 | Development and validation study |
|  |  | incidence | 6 |  | Bhardwaj M et al. 2022 [22] | 9,413 Population from ESTHER cohort study | 50-75 | Comparative evaluation study |
|  |  | incidence | 6 |  | Liao W et al. 2023 [35] | 19,670,000 Asymptomatic adults from English primary care databases (QResearch & CPRD) | 25-84 | Retrospective population-based cohort study |
|  |  | incidence | 6 |  | Tammemägi M et al. 2014 [33] | 208,352 PLCO & NLST Ever-Smokers | 55-74 | Evaluation study |
| PLCOm2012 model | Logistic-regression model | incidence | 6 | 11:  Age; Race; Smoking status; Smoking duration; Smoking intensity; Years since cessation; Personal history of cancer; Family history of lung cancer; Education; BMI; COPD | Feng X et al. 2024 [21] | 240,137 Current/former smokers from 9 European countries (Finland, France, Denmark, Germany, Italy, Spain, Sweden, Netherlands, Norway) | 45-80 | Prospective cohort consortium analysis |
|  |  | incidence | 6 |  | Lebrett M et al. 2020 [37] | 1,429 Ever-Smokers Manchester Lung Health Check (LHC) pilot | 55-74 | Comparison Study |
|  |  | incidence | 6 |  | Ten Haaf K et al. 2017 [32] | 134,124 NLST & PLCO Ever-Smokers | 57-69 | Retrospective validation study |
|  |  | incidence | 6 |  | Gabe R et al. 2024 [40] | 7,826 Ever-Smokers in Yorkshire, UK | 55-80 | Prospective cohort study (randomized controlled trial with community-based LDCT-LCS) |
|  |  | incidence | 6 |  | Jungblut L et al. 2023 [39] | 112 Asymptomatic participants at high risk for lung cancer (55-74 years) | 55-74 | Prospetcive Pilot Study |
|  |  | incidence | 6 |  | Hirsch E et al. 2023 [25] | 48 Ever-Ssmokers | 40-82 | Prospective Pilot Study (NCT03683940) |
|  |  | incidence | 6 |  | Rodriguez A et al. 2024 [26] | 896 High-risk individuals from Boston Medical Center (BMC) database of patients who received LC screening with LDCT between 2015 to 2019 | 57-70 | Retrospective cross-sectional study |
|  |  | incidence | 6 |  | Ostrowski M et al. 2021 [27] | 6,631 Ever-Ssmokers, ≥30 pack-years smoking history | 50-79 | comparative study with cohort data MOLTEST BIS programme |
|  |  | incidence | 6 |  | Pan Z et al. 2023 [36] | 323,344 General population (UK Biobank, UKB) | 37-73 | Development and validation study |
|  |  | incidence | 6 |  | Bhardwaj M et al. 2022 [22] | 9,412 Population from ESTHER cohort study | 50-75 | Comparative evaluation study |
|  |  | incidence | 6 |  | Ngo PJ et al. 2022 [28] | 19,991 Ever-Smokers (current/former) from NSW 45 and Up Study cohort, Australia | 50-80 | Quantitative evaluation using population-based cohort data |
|  |  | incidence | 6 |  | Cleven K et al. 2021 [29] | 3,953 EverSmokers, FDNY-WTC exposed rescue and recovery workers (firefighters/EMS) | 50-80 | Retrospective analysis |
|  |  | incidence | 6 |  | Darling G et al. 2021 [30] | 4,205 Individuals aged 55-74, current/ex-smokers (≥20 pack-years), excluding those with diagnosed lung cancer or under nodule surveillance | 55-74 | Original quantitative evaluation study |
|  |  | incidence | 6 |  | Kats et al. 2021 [38] | 37,327 Ever-Smokers | 55-74 | Retrospective cohort validation study based on EHR |
|  |  | incidence | 6 |  | Bartlett E et al. 2020 [24] | 8,366 Ever-Smokers in West London, UK | 60-75 | Prospective observational pilot study |
|  |  | incidence | 5 |  | Hüsing A et al. 2020 [23] | 16 Mio. projected from 14,834 Ever-Smokers of German population study GEDA 2008-2013 and 20.700 Ever-Smokers from German EPIC cohort (EPIC-D) | 50-79 | Retrospective analysis |
|  |  | incidence | 6 |  | Aggarwal R et al. 2019 [41] | 1,261 Adults aged ≥50 with ≥10 pack-year smoking history, no prior cancer (except nonmelanotic skin), negative baseline LDCT scans | 50-74 | Prospective single-arm cohort study |
|  |  | incidence | 6 |  | Katki H et al. 2018 [13] | 409,726 Ever-Smokers from National Institutes of Health–AARP Diet and Health Study (NIH AARP) and Ever-Smokers from CPS-II (Cancer Prevention Study II) Nutrition Survey cohort | 50-80 | Comparison and Validation Study |
|  |  | incidence | 6 |  | Weber M et al. 2017 [31] | 95,882 Australian ever-smokers from the 45 and Up Study cohort | 45-100 |  |
|  |  | incidence | 6 |  | Wilson D et al. 2015 [34] | 57,108 Current and former smokers (PLuSS and NLST | 50-79 | Original quantitative evaluation of risk prediction models |
|  |  | incidence | 6 |  | Tammemägi M et al. 2013 [20] | 133,580 PLCO and NLST Ever-Smokers | 55-74 | Retrospective modeling study |
|  |  | incidence | 6 |  | Liao W et al. 2023 [35] | 19,670,000 Asymptomatic adults from English primary care databases (QResearch & CPRD) | 25-84 | Retrospective population-based cohort study |
|  |  | incidence | 6 |  | Tammemägi M et al. 2014 [33] | 208,352 PLCO & NLST Ever-smokers | 55-74 | Evaluation study |
| PLCOm2012 model simplified version | Logistic-regression model | incidence | 6 | 6:  Age; Gender; Smoking status; Smoking duration; Smoking intensity; Years since cessation | Ten Haaf K et al. 2017 [32] | 134,124 NLST & PLCO ever-Smokers | 57-69 | Retrospective validation study |
|  |  |  | 6 |  | Tomonaga Y et al. 2023 [49] | 10 Mio Birth cohort 1940-1979 (MISCAN Model), Ever-Smokers 10-40 pack years | 50-85 | Microsimulation-based cost-effectiveness and budget impact analysis based on MISCAN model |
|  |  |  | 6 |  | Toumazis I et al. 2023 [46] | 1,000,000 1960 US birth cohort | 45-90 | Comparative modeling analysis based on four microsimulation models (CISNET) |
|  |  |  | 6 |  | Meza R et al. 2021 [14] | 1,000,000 1950 and 1960 US birth cohort | 45-90 | Comparative simulation modeling study based on four microsimulation models (CISNET) |
|  |  |  | 5 |  | Roseleur J et al. 2024 [48] | 6,700,000 Birth cohort 1945-1969 (5 cohorts) | 54-78 | Microsimulation-based cost-effectiveness and budget impact analysis based on MISCAN model (5 cohorts) |
| PLCOm2012 Race3L | Logistic-regression model | incidence | 6 | 12:  Age; Gender; Race; Smoking status; Smoking duration; Smoking intensity; Years since cessation; Personal history of cancer; Personal history of pneumonia; Chronic bronchitis; Emphysema; BMI | Williams RM et al. 2022 [63] | 41,544 Current/former smokers | 50-80 | Cross-sectional analysis of 2019 Behavioral Risk Factor Surveillance System (BRFSS) data |
| PLCOm2012bu model | Logistic-regression model | incidence | 3 | 11:  Age; Race; Smoking status; Smoking duration; Smoking intensity; Years since cessation; Personal history of cancer; Family history of lung cancer; Education; BMI; COPD | Tammemägi M et al. 2019 [62] | 22,229 Ever-Smokers, NLST participants (LSS and ACRIN subsets) | 57-65 | Secondary analysis of NLST RCT data |
| PLCOm2012noRace model | Logistic-regression model | incidence | 6 | 11:  Age; Race; Smoking status; Smoking duration; Smoking intensity; Years since cessation; Personal history of cancer; Family history of lung cancer; Education; BMI; COPD | Tammemägi M et al. 2024 [52] | 7,768 High-risk individuals in Ontario, Canada | 55-74 | real world case study, multi-center lung cancer screening pilot |
|  |  | incidence | 6 |  | Laisaar T et al. 2025 [53] | 26,759 Individuals aged 55–74 years who had ever smoked | 55-74 | Estonian regional LCS pilot project |
| Polynomial model | Logistic-regression model | incidence | NA | 8:  Age; Gender; Smoking status; Pack Years; Personal history of cancer; Family history of lung cancer; Emphysema; COPD | Maldonado S et al. 2021 [57] | 4,052 Ever-Smokers | 50-69 | Validation Study |
| Safety Net Hospitals (SNH) model | Logistic-regression model | incidence | 6 | 5:  Age; Pack Years; Family history of lung cancer; Emphysema; BMI | Rodriguez A et al. 2024 [26] | 896 High-risk individuals from Boston Medical Center (BMC) database of patients who received LC screening with LDCT between 2015 to 2019 | 57-70 | Retrospective cross-sectional study |
| Spitz 2007 Lung Cancer Risk Measure | Logistic recursively cycling model for relative risks; attributable risk method applied to SEER incidence and mortality rates to obtain baseline rate | incidence | 1 | 14:  Age; Gender; Smoking status; Former smoker; Current smoker; Smoking duration; Smoking intensity; Years since cessation; Asbestos exposure; Dust exposure; No Hay Fever; Personal history of cancer; Family history of lung cancer; Emphysema | Bhardwaj M et al. 2022 [22] | 9,415 Population from ESTHER cohort study | 50-75 | Comparative evaluation study |
|  |  | incidence | 1 |  | Katki H et al. 2018 [13] | 409,726 Ever-Smokers from National Institutes of Health–AARP Diet and Health Study (NIH AARP) and Ever-Smokers from CPS-II (Cancer Prevention Study II) Nutrition Survey cohort | 50-80 | Comparison and Validation Study |
| Two-Stage Clonal Expansion (TSCE) CPS lung cancer death model | Stochastic representation of the cell events | mortality | 1 | 6:  Age; Gender; Smoking status; Smoking duration; Smoking intensity; Years since cessation | Ten Haaf K et al. 2017 [32] | 134,124 NLST & PLCO Ever-Smokers | 57-69 | Retrospective validation study |
| Two-Stage Clonal Expansion (TSCE) lung cancer incidence model | Stochastic representation of the cell events | incidence | 1 |  | Ten Haaf K et al. 2017 [32] | 134,124 NLST & PLCO Ever-Smokers | 57-69 | Retrospective validation study |
| Two-Stage Clonal Expansion (TSCE) NHS/HPFS lung cancer death model | Stochastic representation of the cell events | mortality | 1 |  | Ten Haaf K et al. 2017 [32] | 134,124 NLST & PLCO Ever-Smokers | 57-69 | Retrospective validation study |
| University College London Death (UCLD) model | Machine learning model (ensemble) | mortality | 5 | 3:  Age; Smoking duration; Pack Years | Feng X et al. 2024 [21] | 240,137 Current/former smokers from 9 European countries (Finland, France, Denmark, Germany, Italy, Spain, Sweden, Netherlands, Norway) | 45-80 | Prospective cohort consortium analysis |
|  |  |  | 5 |  | Callender T et al. 2023 [54] | 40,593 PLCO Ever-Smokers | 55-74 | Analysis of data from four prospective cohorts |
| University College London Incidence (UCLI) model | Machine learning model (ensemble) | incidence | 5 | 3:  Age; Smoking duration; Pack Years | Feng X et al. 2024 [21] | 240,137 Current/former smokers from 9 European countries (Finland, France, Denmark, Germany, Italy, Spain, Sweden, Netherlands, Norway) | 45-80 | Prospective cohort consortium analysis |
|  |  |  | 5 |  | Callender T et al. 2023 [54] | 40,593 PLCO Ever-Smokers | 55-74 | Analysis of data from four prospective cohorts |
