# Supplementary material for: Use and impact of risk-based eligibility models in low-dose computed tomography lung cancer screening: a systematic review
Source: Public Health Rev. 2026 Jul 20;47:1609133. doi: 10.3389/phrs.2026.1609133 (PMC13430644; doi:10.3389/phrs.2026.1609133)
Supplement: Supplementary file 2 [file Table2.docx]

**Supplementary Table 2: Results of the assessed models (n = 39) (Risk-based Eligibility Models Review, Europe, 2025).**

| **Model Description** | **Study Description** | | | | **Outcome Description (min / max)** | | | | | | |
| --- | --- | --- | --- | --- | --- | --- | --- | --- | --- | --- | --- |
| **Name of LC Risk-Prediction Model in assessed studies** | **Study first author, year published** | **Study population described in study** | **Age** | **Described study type** | **Discrimination Performance (AUC [95 % CI] or [min-max])** | **Detected LC Cases (n) & Study Context** | **LC Detection Rate ( %)** | **LC deaths averted with risk model (n)** | **LY gained or QALY (n)** | **ICER / QALY** |  |
| Bach model | Feng X et al. 2024 [21] | 240,137 Current/former smokers from 9 European countries (Finland, France, Denmark, Germany, Italy, Spain, Sweden, Netherlands, Norway) | 45-80 | Prospective cohort consortium analysis | 0.70 (0.68-0.72) Finland to 0.81 (0.76-0.85) Germany [min-max] | Expected n = 63.2 expected vs. observed n = 40 (Spain) toexpected n = 496.2 vs. observed n= 654 (Finland) [min-max] | NR | NR | NR | NR |  |
|  | Ten Haaf K et al. 2017 [32] | 134,124 NLST & PLCO Ever-Smokers | 57-69 | Retrospective validation study | 0.68- 0.79 [min-max] | NR | NR | NR | NR | NR |  |
|  | Ostrowski M et al. 2021 [27] | 6,631 Ever-Smokers, ≥30 pack-years smoking history | 50-79 | comparative study with cohort data MOLTEST BIS programme | 0.701 (0.655-0.748) | n = 69 of 1,313 screened | 5.3 % | NR | NR | NR |  |
|  | Bhardwaj M et al. 2022 [22] | 9,407 Population from ESTHER cohort study | 50-75 | Comparative evaluation study | 0.766- 0.782 [min-max] | n = 73-83 (11.8 % – 14.7 % more cases vs. LDCT trial criteria) | 71.6 % - 81.4 % [min-max] | NR | NR | NR |  |
|  | Meza R et al. 2021 [14] | 1,000,000 1950 and 1960 US birth cohort | 45-90 | Comparative simulation modeling study based on four microsimulation models (CISNET) | NR | NR | NR | n = 355- 662 ( 69 Bach model scenarios) | n = 4,017-8,387 LY gained (69 Bach model scenarios) | NR |  |
|  | Cleven K et al. 2021 [29] | 3,953 Ever-Smokers, FDNY-WTC exposed rescue and recovery workers (firefighters/EMS) | 50-80 | Retrospective analysis | NR | n = 11 of 29 (37.9 %) | 7 % (n = 29 of 395) | NR | NR | NR |  |
|  | Hüsing A et al. 2020 [23] | 16 Mio. projected from 14,834 Ever-Smokers of German population study GEDA 2008-2013 and 20.700 Ever-Smokers from German EPIC cohort (EPIC-D) | 50-79 | Retrospective analysis | 0.70-0.81 [min-max] | n = 14.000-28.700 [min-max] across 3 eligibility groups, re-calibrated | 37 %-75 % [min-max] across 3 eligibility groups, re-calibrated | NR | NR | NR |  |
|  | Katki H et al. 2018 [13] | 409,726 Ever-Smokers from National Institutes of Health–AARP Diet and Health Study (NIH AARP) and Ever-Smokers from CPS-II (Cancer Prevention Study II) Nutrition Survey cohort | 50-80 | Comparison and Validation Study | 0.755 (0.752-0.759) vs. 0.750 (0.740-0.760) | n = 320.37 vs. 283.39 cases per 100.000/year) | NR | NR | NR | NR |  |
|  | Wilson D et al. 2015 [34] | 57,108 Current and former smokers (PLuSS and NLST | 50-79 | Original quantitative evaluation of risk prediction models | 0.687-0.710 [min-max] | NR | NR | NR | NR | NR |  |
|  | Bach P et al. 2003 [42] | 18,314 Heavy smoker and asbestos cohort | 44-75 | modeling study based on CARET cohort study | 0.72 (C-Index) | NR | 5.90 % | NR | NR | NR |  |
|  | Liao W et al. 2023 [35] | 19,670,000 Asymptomatic adults from English primary care databases (QResearch & CPRD) | 25-84 | Retrospective population-based cohort study | 0.575 (0.566-0.585) women, 0.586 (0.577-0.595) men | NR | NR | NR | NR | NR |  |
| CanPredict (lung model), 10 years | Liao W et al. 2023 [35] | 19,670,000 Asymptomatic adults from English primary care databases (QResearch & CPRD) | 25-84 | Retrospective population-based cohort study | 0.724 (0.715-0.733) women, 0.731 (0.723-0.738) men | NR | NR | NR | NR | NR |  |
| CanPredict (lung model), 5 years | Liao W et al. 2023 [35] | 19,670,000 Asymptomatic adults from English primary care databases (QResearch & CPRD) | 25-84 | Retrospective population-based cohort study | 0.727 (0.715-0.739) women, 0.735 (0.726-0.745) men | n = 4,542 (Strategy 1B QResearch validation cohort) | 87.7 % of 5,180 (Strategy 1B QResearch validation cohort) | NR | NR | NR |  |
| CanPredict (lung model), 6 years | Liao W et al. 2023 [35] | 19,670,000 Asymptomatic adults from English primary care databases (QResearch & CPRD) | 25-84 | Retrospective population-based cohort study | 0.726 (0.715-0.737) women, 0.735 (0.726-0.743) men | n = 4,069 (Strategy 2B QResearch validation cohort) | 66.8 % of 6,093 (Strategy 2B QResearch validation cohort) | NR | NR | NR |  |
| Computable phenotype (CP) algorithms for LCS eligibility, Rule-based algorithm combining structured EHR data and NLP-processed unstructured data | Yang S et al. 2025 [55] | 5,778 Individuals from University of Florida Health Integrated Data Repository (2012–2022 LDCT recipients) | 50-80 | Original quantitative evaluation study | F1 Score: 0.70-0.84 [min-max] | NR | NR | NR | NR | NR |  |
| COSMOS model | Maisonneuve P et al. 2023 [56] | 5,203 Asymptomatic individuals aged ≥50, heavy smokers (≥20 pack-years) | 50-84 | Prospective cohort study (based on the COSMOS trial) | 0.759 | n = 161 of 18,095 | 1.6 % over 5 years | NR | NR | NR |  |
| Hoggart Model | Bhardwaj M et al. 2022 [22] | 9,408 Population from ESTHER cohort study | 50-75 | Comparative evaluation study | 0.705–0.712 [min-max] | NR | NR | NR | NR | NR |  |
|  | Katki H et al. 2018 [13] | 409,726 Ever-Smokers from National Institutes of Health–AARP Diet and Health Study (NIH AARP) and Ever-Smokers from CPS-II (Cancer Prevention Study II) Nutrition Survey cohort | 50-80 | Comparison and Validation Study | 0.714 (0.699 - 0.728) vs.0.751 (0.722 - 0.781) | n = 275.65 vs. 317.95 cases per 100.000/year | NR | NR | NR | NR |  |
| HUNT model (Norwegian Nord-Trøndelag Health Study model) | Feng X et al. 2024 [21] | 240,137 Current/former smokers from 9 European countries (Finland, France, Denmark, Germany, Italy, Spain, Sweden, Netherlands, Norway) | 45-80 | Prospective cohort consortium analysis | 0.70 (0.68-0.72) Finland to 0.81 (0.78 0.85) Germany [min-max] | Expected n = 50.9 vs. observed n = 41 Spain to expected n = 453.7 vs. observed n = 830 Finland [min-max] | NR | NR | NR | NR |  |
|  | Roe O D et al. 2019 [50] | 4,051 DLST cohort participants | 50-70 | Retrospective analysis of DLCST cohort data | C-index 0.783 (internal) vs. 0.663 (external) | n = 148 | 3.77 % (PPV) | NR | NR | NR |  |
| Knoke model | Ten Haaf K et al. 2017 [32] | 134,124 NLST & PLCO Ever-Smokers | 57-69 | Retrospective validation study | 0.66-0.78 [min-max] | NR | NR | NR | NR | NR |  |
| LCDRAT (Lung Cancer Death Risk Assessment Tool) | Feng X et al. 2024 [21] | 240,137 Current/former smokers from 9 European countries (Finland, France, Denmark, Germany, Italy, Spain, Sweden, Netherlands, Norway) | 45-80 | Prospective cohort consortium analysis | 0.68 (0.59-0.77) Netherlands to 0.83 (0.78-0.89) Germany [min-max] | Expected n = 28.9 vs. observed n = 23 Norway to expected n = 396.9 vs. observed 414 Finland [min-max] | NR | NR | NR | NR |  |
|  | Katki H et al. 2016 [43] | 132,160 PLCO & NLST Ever-Smokers | 55-74 | Development and Validitation Study | 0.76-0.83 [min-max] | 370.6-475.5 [min-max] | NR | NR | NR | NR |  |
|  | Toumazis I et al. 2023 [46] | 1,000,000 1960 US birth cohort | 45-90 | Comparative modeling analysis based on four microsimulation models (CISNET) | 0.78 | NR | NR | NR | 1.883 | 97,284 (base case analysis) |  |
|  | Bhardwaj M et al. 2022 [22] | 9,411 Population from ESTHER cohort study | 50-75 | Comparative evaluation study | (0.765–0.787) [min-max] | n = 74-86 (10.8 %-15.7 % more cases vs. LDCT criteria) | 72.5 % - 84.3 % [min-max] | NR | NR | NR |  |
|  | Meza R et al. 2021 [14] | 1,000,000 1950 and 1960 US birth cohort | 45-90 | Comparative simulation modeling study based on four microsimulation models (CISNET) | NR | NR | NR | n = 348-655 (27 MLCDRAT scenarios) | n = 3,940-7,830 LY gained (27 MLCDRAT scenarios) | NR |  |
|  | Katki H et al. 2018 [13] | 409,726 Ever-Smokers from National Institutes of Health–AARP Diet and Health Study (NIH AARP) and Ever-Smokers from CPS-II (Cancer Prevention Study II) Nutrition Survey cohort | 50-80 | Comparison and Validation Study | 0.779 (0.776 - 0.782) vs. 0.786 (0.770 - 0.802) | n = 173.92 vs. 179.16 cases per 100.000/year | NR | NR | NR | NR |  |
| LCRAT (Lung Cancer Incidence Model) | Feng X et al. 2024 [21] | 240,137 Current/former smokers from 9 European countries (Finland, France, Denmark, Germany, Italy, Spain, Sweden, Netherlands, Norway) | 45-80 | Prospective cohort consortium analysis | 0.71 (0.70-0.73) Finland to 0.80 (0.75 0.85) Germany [min-max] | Expected n = 51.6 vs. observed n = 40 Spain to expected n = 641.1 vs. observed n = 654 Finland [min-max] | NR | NR | NR | NR |  |
|  | Katki H et al. 2016 [43] | 132,160 PLCO & NLST Ever-Smokers | 55-74 | Development and Validitation Study | 0.69-0.81 [min-max] | n = 588.5-763.8 [min-max] | NR | 62382 | NR | NR |  |
|  | Bhardwaj M et al. 2022 [22] | 9,410 Population from ESTHER cohort study | 50-75 | Comparative evaluation study | 0.771-0.786) [min-max] | n = 74-87 (11.8 %-17.6 % more cases vs. LDCT criteria) | 72.5 % - 85.3 % [min-max] | NR | NR | NR |  |
|  | Kats et al. 2021 [38] | 37,327 Ever Smokers | 55-74 | Retrospective cohort validation study based on EHR | 0.72 (0.70-0.74) | NA | NR | NR | NR | NR |  |
|  | Hüsing A et al. 2020 [23] | 16 Mio. projected from 14,834 Ever-Smokers of German population study GEDA 2008-2013 and 20.700 Ever-Smokers from German EPIC cohort (EPIC-D) | 50-79 | Retrospective analysis | 0.70-0.80 [min-max] | n = 16.700-29.400 [min-max] across 3 eligibility groups, re-calibrated | 44 %-77 % [min-max] across 3 eligibility groups, re-calibrated | NR | NR | NR |  |
|  | Katki H et al. 2018 [13] | 409,726 Ever-Smokers from National Institutes of Health–AARP Diet and Health Study (NIH AARP) and Ever-Smokers from CPS-II (Cancer Prevention Study II) Nutrition Survey cohort | 50-80 | Comparison and Validation Study | 0.771 (0.768 - 0.775) vs. 0.770 (0.757 - 0.784) | n = 302.03 vs. 304.13 cases per 100.000/year | NR | NR | NR | NR |  |
|  | Liao W et al. 2023 [35] | 19,670,000 Asymptomatic adults from English primary care databases (QResearch & CPRD) | 25-84 | Retrospective population-based cohort study | 0.642 (0.629-0.655) women, 0.657 (0.646-0.667) men | NR | NR | NR | NR | NR |  |
| LCRAT+CT | Maldonado S et al. 2021 [57] | 4,052 Ever-Smokers | 50-69 | Validation Study | 0.73 (0.63-0.82) | NR | 0.009%-2.76% [min-max] | NR | NR | NR |  |
| Liverpool Lung Project (LLP) model | Ten Haaf K et al. 2017 [32] | 134,124 NLST & PLCO Ever-Smokers | 57-69 | Retrospective validation study | 0.65-0.76 [min-max] | NR | NR | NR | NR | NR |  |
|  | Ostrowski M et al. 2021 [27] | 6,631 Ever-Smokers, ≥30 pack-years smoking history | 50-79 | comparative study with cohort data MOLTEST BIS programme | 0.667 (0.619–0.714) | n = 114 of 3,336 screened | 3.4 % | NR | NR | NR |  |
|  | Bhardwaj M et al. 2022 [22] | 9,409 Population from ESTHER cohort study | 50-75 | Comparative evaluation study | 0.676- 0.744 [min-max] | NR | NR | NR | NR | NR |  |
|  | Hüsing A et al. 2020 [23] | 16 Mio. projected from 14,834 Ever-Smokers of German population study GEDA 2008-2013 and 20.700 Ever-Smokers from German EPIC cohort (EPIC-D) | 50-79 | Retrospective analysis | 0.75-0.75 [min-max] | n = 15.900-29.700 [min-max] across 3 eligibility groups, re-calibrated | 42 %-78 % [min-max] across 3 eligibility groups, re-calibrated | NR | NR | NR |  |
|  | Katki H et al. 2018 [13] | 409,726 Ever-Smokers from National Institutes of Health–AARP Diet and Health Study (NIH AARP) and Ever-Smokers from CPS-II (Cancer Prevention Study II) Nutrition Survey cohort | 50-80 | Comparison and Validation Study | 0.726 (0.722 - 0.731) vs. 0.726 (0.711 - 0.740) | n = 302.03 vs. 304.13 cases of 100.000/year | NR | NR | NR | NR |  |
|  | Raji O et al. 2012 [44] | 2,922 EUELC (European), Harvard (North American), and LLPC (UK) studie | 40-79 | Case-control & prospective cohort | 0.67-0.82 [min-max] | n = 420 cases of 7,652 in LLPC cohort | 5.5 % | NR | NR | NR |  |
|  | Cassidy A et al. 2007 [45] | 1,736 Residents of the Liverpool area, incident cases of histologically or cytologically confirmed lung cancer were between 20 and 80 years of age | 57-75 | Case-control study | 0.71 (original); 0.70 (cross-validation) | NR | NR | NR | NR | NR |  |
| Liverpool Lung Project (LLP) model simplified version | Ten Haaf K et al. 2017 [32] | 134,124 NLST & PLCO Ever-Smokers | 57-69 | Retrospective validation study | 0.65–0.75 [min-max] | NR | NR | NR | NR | NR |  |
| Liverpool Lung Project (LLP) model version 2 | Feng X et al. 2024 [21] | 240,137 Current/former smokers from 9 European countries (Finland, France, Denmark, Germany, Italy, Spain, Sweden, Netherlands, Norway) | 45-80 | Prospective cohort consortium analysis | 0.64 (0.57-0.72) Norway to 0.78 (0.74-0.82) Sweden [min-max] | Expected n = 91.9 vs. observed n = 40 Spain to expected n = 574.5 vs. n = 654 Finland [min-max] | NR | NR | NR | NR |  |
|  | Lebrett M et al. 2020 [37] | 1,429 Ever-Smokers Manchester Lung Health Check (LHC) pilot | 55-74 | Comparison Study | NR | NR | 93.5 % (n=58/62) | NR | NR | NR |  |
|  | Gabe R et al. 2024 [40] | 7,826 Ever-Smokers in Yorkshire, UK | 55-80 | Prospective cohort study (randomized controlled trial with community-based LDCT-LCS) | 0.81 | n = 217 (188.9-245.1) | 5.53 % (n=217/3921 screened) | NR | NR | NR |  |
|  | Field J K et al. 2021 [47] | 75,958 Risk population | 50-79 | Case-control and prospective cohort designs | 0.81 (0.79-0.82) | NR | NR | NR | NR | NR |  |
|  | Bartlett E et al. 2020 [24] | 8,366 Ever-Smokers in West London, UK | 60-75 | Prospective observational pilot study | NR | n = 29 within risk score range 2.97–35.92 % | 2.7% within risk score range 2.97–35.92 % | NR | NR | NR |  |
|  | Liao W et al. 2023 [35] | 19,670,000 Asymptomatic adults from English primary care databases (QResearch & CPRD) | 25-84 | Retrospective population-based cohort study | 0.647 (0.635-0.659) women, 0.655 (0.645-0.665) men | n = 4,229 (Strategy 1A QResearch validation cohort) | 81.6% of 5,180 (Strategy 1A QResearch validation cohort) | NR | NR | NR |  |
| Liverpool Lung Project (LLP) model version 3 | Feng X et al. 2024 [21] | 240,137 Current/former smokers from 9 European countries (Finland, France, Denmark, Germany, Italy, Spain, Sweden, Netherlands, Norway) | 45-80 | Prospective cohort consortium analysis | 0.64 (0.57-0.72) Norway to 0.78 (0.73-0.82) Germany [min-max] | Expected n = 44.3 vs. observed n = 40 Spain to expected n = 269.3 vs. observed n = 654 Finland [min-max] | NR | NR | NR | NR |  |
|  | Pan Z et al. 2023 [36] | 323,344 General population (UK Biobank, UKB) | 37-73 | Development and validation study | 0.829-0.846 general population / 0.657-0.805 Ever-Smokers | NR | NR | NR | NR | NR |  |
|  | Bhardwaj M et al. 2022 [22] | 9,417 Population from ESTHER cohort study | 50-75 | Comparative evaluation study | 0.725- 0.773 [min-max] | NR | NR | NR | NR | NR |  |
|  | Field J K et al. 2021 [47] | 75,958 Risk population | 50-79 | Case-control and prospective cohort designs | 0.81 (0.79-0.82) | n = 599 | NR | NR | NR | NR |  |
|  | Liao W et al. 2023 [35] | 19,670,000 Asymptomatic adults from English primary care databases (QResearch & CPRD) | 25-84 | Retrospective population-based cohort study | 0.660 (0.648-0.672) women, 0.662 (0.652-0.672) men | NR | NR | NR | NR | NR |  |
| Liverpool Lung Project (LLPi) incidence model | Bhardwaj M et al. 2022 [22] | 9,416 Population from ESTHER cohort study | 50-75 | Comparative evaluation study | 0.725- 0.773 [min-max] | NR | NR | NR | NR | NR |  |
|  | Katki H et al. 2018 [13] | 409,726 Ever-Smokers from National Institutes of Health–AARP Diet and Health Study (NIH AARP) and Ever-Smokers from CPS-II (Cancer Prevention Study II) Nutrition Survey cohort | 50-80 | Comparison and Validation Study | 0.714 (0.711 - 0.717) vs. 0.721 (0.711 - 0.732) | n = 314.86 vs. 510.94 cases per 100.000/year | NR | NR | NR | NR |  |
| Lung cancer screening decision (ENGAGE) tool | Toumazis I et al. 2020 [58] | Subpopulation of Ever-Smokers | 50-80 | Original quantitative evaluation study using partially observable Markov decision process (POMDP) | NR | NR | NR | 13.18 QALYs per person at age 50 (13.17-13.18) | NR | NR |  |
| Lung-cancer Death Risk Measure | Katki H et al. 2018 [13] | 409,726 Ever-Smokers from National Institutes of Health–AARP Diet and Health Study (NIH AARP) and Ever-Smokers from CPS-II (Cancer Prevention Study II) Nutrition Survey cohort | 50-80 | Comparison and Validation Study | 0.773 (0.769 - 0.777) vs. 0.774 (0.757 - 0.790) | n = 173.92 vs. 179.16 cases per 100.000/year | NR | NR | NR | NR |  |
|  | Kovalchik S et al. 2013 [51] | 53,158 LDCT Group & Radiography Group | 55-74 | Modeling Study | C-statistic, 0.80 (0.77 -0.82) | NR | NR | NR | NR | NR |  |
| LungFlag model | Trujillo J C et al. 2025 [59] | 3,835,128 Individuals meeting USPSTF 2013 criteria in Spain (adults aged 55-80 with ≥30 pack-year smoking history) | 55-80 | Cost-effectiveness analysis using decision-tree + Markov model | NR | NR | 75 % early-stage (0-II) vs 19.1 % in non-screening | +97,612 QALYs vs non-screening | 61.66 Mio. QALYs vs 61.15 Mio. non-screening | ICER: €72,252/QALY (LungFlag model dominant over non-screening |  |
| Medial EarlySign (MES) machine learning model | Gould M et al. 2021 [60] | 196,102 NSCLC case patients | 45-90 | Retrospective cohort study, Kaiser Permanente Southern California (KPSC) | 0.856 (0.841- 0.871) | NR | NR | NR | NR | NR |  |
| OWL (Optimized Early Warning Model for Lung Cancer Risk) model | Feng X et al. 2024 [21] | 240,137 Current/former smokers from 9 European countries (Finland, France, Denmark, Germany, Italy, Spain, Sweden, Netherlands, Norway) | 45-80 | Prospective cohort consortium analysis | 0.70 (0.69-0.72) Finland to 0.82 (0.77-0.86) Germany [min-max] | Expected n = 42.4 vs. observed n = 40 Spain to expected n = 458.9 vs. observed n = 654 Finland [min-max] | NR | NR | NR | NR |  |
|  | Pan Z et al. 2023 [36] | 323,344 General population (UK Biobank, UKB) | 37-73 | Development and validation study | 0.849-0.865 general population / 0.711-0.855 Ever-Smokers | NR | NR | NR | NR | NR |  |
| Pan-Canadian Early Detection of Lung Cancer (PanCan) Model | Tammemägi M et al. 2017 [61] | 7,044 Ever-Smokers aged 50–75 years without lung cancer history, recruited across eight Canadian centers | 50-75 | Singe-arm, prospective study | 0.783 (0.770–0.801) | n = 172 of 164 individuals | 6.78 % (164/2537) cumulative | NR | NR | NR |  |
| Pittsburgh Predictor | Bhardwaj M et al. 2022 [22] | 9,414 Population from ESTHER cohort study | 50-75 | Comparative evaluation study | 0.763-0.778 [min-max] | NR | NR | NR | NR | NR |  |
|  | Katki H et al. 2018 [13] | 409,726 Ever-Smokers from National Institutes of Health–AARP Diet and Health Study (NIH AARP) and Ever-Smokers from CPS-II (Cancer Prevention Study II) Nutrition Survey cohort | 50-80 | Comparison and Validation Study | 0.752 (0.748-0.757) vs. 0.747 (0.731-0.762) | n = 303.56 vs. 297.91 cases per 100.000/year | NR | NR | NR | NR |  |
|  | Wilson D et al. 2015 [34] | 57,108 Current and former smokers (PLuSS and NLST | 50-79 | Original quantitative evaluation of risk prediction models | 0.678-0.701 [min-max] | NR | NR | NR | NR | NR |  |
|  | Liao W et al. 2023 [35] | 19,670,000 Asymptomatic adults from English primary care databases (QResearch & CPRD) | 25-84 | Retrospective population-based cohort study | 0.643 (0.631 - 0.654) women, 0.657 (0.648 - 0.666) men | NR | NR | NR | NR | NR |  |
| PLCO2012 result model | Tammemägi M et al. 2019 [62] | 22,229 Ever-Smokers, NLST participants (LSS and ACRIN subsets) | 57-65 | Secondary analysis of NLST RCT data | 0.687 (0.645-0.728) | NR | NR | NR | NR | NR |  |
| PLCOall2014 | Pan Z et al. 2023 [36] | 323,344 General population (UK Biobank, UKB) | 37-73 | Development and validation study | 0.807-0.869 general population / 0.710-0.806 Ever-Smokers | NR | NR | NR | NR | NR |  |
|  | Bhardwaj M et al. 2022 [22] | 9,413 Population from ESTHER cohort study | 50-75 | Comparative evaluation study | 0.741-0.749 [min-max] | NR | NR | NR | NR | NR |  |
|  | Liao W et al. 2023 [35] | 19,670,000 Asymptomatic adults from English primary care databases (QResearch & CPRD) | 25-84 | Retrospective population-based cohort study | 0.526 (0.513 - 0.540) women, 0.540 (0.529 - 0.551) men  0,526 women / 0,540 men | NR | NR | NR | NR | NR |  |
|  | Tammemägi M et al. 2014 [33] | 208,352 PLCO & NLST Ever-Smokers | 55-74 | Evaluation study | 0.833- 0.872 [min-max] | NR | NR | NR | NR | NR |  |
| PLCOm2012 model | Feng X et al. 2024 [21] | 240,137 Current/former smokers from 9 European countries (Finland, France, Denmark, Germany, Italy, Spain, Sweden, Netherlands, Norway) | 45-80 | Prospective cohort consortium analysis | 0.70 (0.68-0.72) to 0.81 (0.77-0.84) [min-max] | Expected n = 34.9 vs. observed n = 41 Spain toexpected n = 584 vs. observed n = 830 Finland [min-max] | NR | NR | NR | NR |  |
|  | Lebrett M et al. 2020 [37] | 1,429 Ever-Smokers Manchester Lung Health Check (LHC) pilot | 55-74 | Comparison Study | NR | n = 19-42 [min-max] | NR | NR | NR | NR |  |
|  | Ten Haaf K et al. 2017 [32] | 134,124 NLST & PLCO Ever-Smokers | 57-69 | Retrospective validation study | 0.69-0.80 [min-max] | NR | NR | NR | NR | NR |  |
|  | Gabe R et al. 2024 [40] | 7,826 Ever-Smokers in Yorkshire, UK | 55-80 | Prospective cohort study (randomized controlled trial with community-based LDCT-LCS) | 0.82 | n = 257 (226,3-287,7) | 4.77% (257/5389 screened) | NR | NR | NR |  |
|  | Jungblut L et al. 2023 [39] | 112 Asymptomatic participants at high risk for lung cancer (55-74 years) | 55-74 | Prospetcive Pilot Study | NR | n = 4 | 3.6% (1.0-12.1%) | NR | NR | NR |  |
|  | Hirsch E et al. 2023 [25] | 48 Ever-Smokers | 40-82 | Prospective Pilot Study (NCT03683940) | NR | n = 2 of 48 | 4.17 % | NR | NR | NR |  |
|  | Rodriguez A et al. 2024 [26] | 896 High-risk individuals from Boston Medical Center (BMC) database of patients who received LC screening with LDCT between 2015 to 2019 | 57-70 | Retrospective cross-sectional study | NR | NR | NR | NR | NR | NR |  |
|  | Ostrowski M et al. 2021 [27] | 6,631 Ever-Smokers, ≥30 pack-years smoking history | 50-79 | comparative study with cohort data MOLTEST BIS programme | 0.717 (0.670–0.763) | n = 150 of 5,470 (97.4%) | 2.7 |  |  |  |  |
|  | Pan Z et al. 2023 [36] | 323,344 General population (UK Biobank, UKB) | 37-73 | Development and validation study | NA / 0.713-0.807 Ever-Smokers | NR | NR | NR | NR | NR |  |
|  | Bhardwaj M et al. 2022 [22] | 9,412 Population from ESTHER cohort study | 50-75 | Comparative evaluation study | 0.739- 0.751 [min-max] | NR | NR | NR | NR | NR |  |
|  | Ngo PJ et al. 2022 [28] | 19,991 Ever-Smokers (current/former) from NSW 45 and Up Study cohort, Australia | 50-80 | Quantitative evaluation using population-based cohort data | NR | NR | NR | NR | NR | NR |  |
|  | Cleven K et al. 2021 [29] | 3,953 Ever-Smokers, FDNY-WTC exposed rescue and recovery workers (firefighters/EMS) | 50-80 | Retrospective analysis | NR | n = (13/32; 40.6%) | 8 % (32/409 screened) | NR | NR | NR |  |
|  | Darling G et al. 2021 [30] | 4,205 Individuals aged 55-74, current/ex-smokers (≥20 pack-years), excluding those with diagnosed lung cancer or under nodule surveillance | 55-74 | Original quantitative evaluation study | NR | n = 28 (1.7% of 1,624 baseline scans) | 1.7 % | NR | NR | NR |  |
|  | Kats et al. 2021 [38] | 37,327 Ever-Smokers | 55-74 | Retrospective cohort validation study based on EHR | 0.71 (0.69-0.73) | n = 695 vs. 639 NLST criteria | 8.8 % vs. NLST criteria | NR | NR | NR |  |
|  | Bartlett E et al. 2020 [24] | 8,366 Ever-Smokers in West London, UK | 60-75 | Prospective observational pilot study | NR | n = 29 within risk score range 1.04 to 25.3 % | 2.7 % within risk score range 1.04 to 25.3 % | NR | NR | NR |  |
|  | Hüsing A et al. 2020 [23] | 16 Mio. projected from 14,834 Ever-Smokers of German population study GEDA 2008-2013 and 20.700 Ever-Smokers from German EPIC cohort (EPIC-D) | 50-79 | Retrospective analysis | 0.68-0.80 [min-max] | n = 20.100-32.500 [min-max] across 3 eligibility groups, re-calibrated | 52 %-85 % [min-max] across 3 eligibility groups, re-calibrated | NR | NR | NR |  |
|  | Aggarwal R et al. 2019 [41] | 1,261 Adults aged ≥50 with ≥10 pack-year smoking history, no prior cancer (except nonmelanotic skin), negative baseline LDCT scans | 50-74 | Prospective single-arm cohort study | NR | n = 17 rescreened cohort | 1.6 %-11 % [min-max] | NR | NR | NR |  |
|  | Katki H et al. 2018 [13] | 409,726 Ever-Smokers from National Institutes of Health–AARP Diet and Health Study (NIH AARP) and Ever-Smokers from CPS-II (Cancer Prevention Study II) Nutrition Survey cohort | 50-80 | Comparison and Validation Study | 0.769 (0.766 - 0.772) vs. 0.754 (0.741 - 0.767) | n = 303.56 vs. 297.91 cases per 100.000/year | NR | NR | NR | NR |  |
|  | Weber M et al. 2017 [31] | 95,882 Australian Ever-Smokers from the 45 and Up Study cohort | 45-100 |  | 0.80 (0.78–0.81) | n = 724 |  | NR | NR | NR |  |
|  | Wilson D et al. 2015 [34] | 57,108 Current and former smokers (PLuSS and NLST | 50-79 | Original quantitative evaluation of risk prediction models | 0.690-0.721 [min-max] | NR | NR | NR | NR | NR |  |
|  | Tammemägi M et al. 2013 [20] | 133,580 PLCO and NLST Ever-Smokers | 55-74 | Retrospective modeling study | 0.803 | NR | NR | NR | NR | NR |  |
|  | Liao W et al. 2023 [35] | 19,670,000 Asymptomatic adults from English primary care databases (QResearch & CPRD) | 25-84 | Retrospective population-based cohort study | 0.531 (0.517 - 0.544) women, 0.545 (0.534–0.557) men | n = 2,583 (Strategy 2A QResearch validation cohort) | 42.4 % of 6,093 (Strategy 2A QResearch validation cohort) | NR | NR | NR |  |
|  | Tammemägi M et al. 2014 [33] | 208,352 PLCO & NLST Ever-Smokers | 55-74 | Evaluation study | 0.782- 0.813 [min-max] | NR | NR | NR | NR | NR |  |
| PLCOm2012 model simplified version | Ten Haaf K et al. 2017 [32] | 134,124 NLST & PLCO Ever-Smokers | 57-69 | Retrospective validation study | 0.68- 0.79 | NR | NR | NR | NR | NR |  |
|  | Tomonaga Y et al. 2023 [49] | 10 Mio Birth cohort 1940-1979 (MISCAN Model), Ever-Smokers 10-40 pack years | 50-85 | Microsimulation-based cost-effectiveness and budget impact analysis based on MISCAN model | NR | NR | NR | NR | n = 1,305-3,504 [min-max] | € 20,884 ACER (average cost-effectiveness ratio) |  |
|  | Toumazis I et al. 2023 [38] | 1,000,000 1960 US birth cohort | 45-90 | Comparative modeling analysis based on four microsimulation models (CISNET) | 0.784 | n = 1427 | NR | n = 493 | 1,857 | € 94,659 |  |
|  | Meza R et al. 2021 [14] | 1,000,000 1950 and 1960 US birth cohort | 45-90 | Comparative simulation modeling study based on four microsimulation models (CISNET) | NR | NR | NR | n = 356-635 (48 MPLCOm2012 scenarios) | n = 4,147-8,164 LY gained  (48 MPLCOm2012scenarios) | NR |  |
|  | Roseleur J et al. 2024 [48] | 6,700,000 Birth cohort 1945-1969 (5 cohorts) | 54-78 | Microsimulation-based cost-effectiveness and budget impact analysis based on MISCAN model (5 cohorts) | NR | NR | NR | n = 62-95 per 100.000 [min-max] | 243- 386 QALY per 100,000 [min-max] | AUD 58,774 - AUD 62,754 [min-max] |  |
| PLCOm2012 Race3L | Williams RM et al. 2022 [63] | 41,544 Current/former smokers | 50-80 | Cross-sectional analysis of 2019 Behavioral Risk Factor Surveillance System (BRFSS) data | 0.72-0.82 | NR | 13.2 % | NR | NR | NR |  |
| PLCOm2012bu model | Tammemägi M et al. 2019 [62] | 22,229 Ever-Smokers, NLST participants (LSS and ACRIN subsets) | 57-65 | Secondary analysis of NLST RCT data | 0.761 (0.716-0.799) | n = 298 of 22 229 individuals | 1.3 % | NR | NR | NR |  |
| PLCOm2012noRace model | Tammemägi M et al. 2024 [52] | 7,768 High-risk individuals in Ontario, Canada | 55-74 | real world case study, multi-center lung cancer screening pilot | 0.946 Youden’s J index | n = 106 of 4451 (2.4%, 95% CI 2.0–2.9%) | 2.2% (1.8–2.7%) | NR | NR | NR |  |
|  | Laisaar T et al. 2025 [53] | 26,759 Individuals aged 55–74 years who had Ever smoked | 55-74 | Estonian regional LCS pilot project | NA | n = 25 of 2304 | 1.09 % (0.70–1.60 %) vs. 0.83 % (0.55–1.21) | NR | NR | NR |  |
| Polynomial model | Maldonado S et al. 2021 [57] | 4,052 Ever-Smokers | 50-69 | Validation Study | 0.75 (0.67-0.83) |  | NR | NR | NR | NR |  |
| Safety Net Hospitals (SNH) model | Rodriguez A et al. 2024 [26] | 896 High-risk individuals from Boston Medical Center (BMC) database of patients who received LC screening with LDCT between 2015 to 2019 | 57-70 | Retrospective cross-sectional study | NR | NR | NR | NR | NR | NR |  |
| Spitz 2007 Lung Cancer Risk Measure | Bhardwaj M et al. 2022 [22] | 9,415 Population from ESTHER cohort study | 50-75 | Comparative evaluation study | 0.638-0.725 [min-max] | NR | NR | NR | NR | NR |  |
|  | Katki H et al. 2018 [13] | 409,726 Ever-Smokers from National Institutes of Health–AARP Diet and Health Study (NIH AARP) and Ever-Smokers from CPS-II (Cancer Prevention Study II) Nutrition Survey cohort | 50-80 | Comparison and Validation Study | 0.707 (0.700 - 0.714) vs. 0.624 (0.588 - 0.661) | n = 275.65 vs. 317.95 cases per 100.000/year | NR | NR | NR | NR |  |
| Two-Stage Clonal Expansion (TSCE) CPS lung cancer death model | Ten Haaf K et al. 2017 [32] | 134,124 NLST & PLCO Ever-Smokers | 57-69 | Retrospective validation study | 0.61- 0.75 [min-max] | NR | NR | NR | NR | NR |  |
| Two-Stage Clonal Expansion (TSCE) lung cancer incidence model | Ten Haaf K et al. 2017 [32] | 134,124 NLST & PLCO Ever-Smokers | 57-69 | Retrospective validation study | 0.67- 0.79 [min-max] | NR | NR | NR | NR | NR |  |
| Two-Stage Clonal Expansion (TSCE) NHS/HPFS lung cancer death model | Ten Haaf K et al. 2017 [32] | 134,124 NLST & PLCO Ever-Smokers | 57-69 | Retrospective validation study | 0.67- 0.78 [min-max] | NR | NR | NR | NR | NR |  |
| University College London Death (UCLD) model | Feng X et al. 2024 [21] | 240,137 Current/former smokers from 9 European countries (Finland, France, Denmark, Germany, Italy, Spain, Sweden, Netherlands, Norway) | 45-80 | Prospective cohort consortium analysis | 0.69 (0.60 - 0.78) EPIC The Netherlands - 0.82 (0.77 - 0.87) EPIC Germany | Expected n = 31.6 vs. observed n = 23 Norway to expected n = 271.3 vs. observed n = 414 Finland [min-max] | NR | NR | NR | NR |  |
|  | Callender T et al. 2023 [54] | 40,593 PLCO Ever-Smokers | 55-74 | Analysis of data from four prospective cohorts | 0.803 (0.783-0.824) | NR | Brier score 0.0084 (0.0075-0.0093) | NR | NR | NR |  |
| University College London Incidence (UCLI) model | Feng X et al. 2024 [21] | 240,137 Current/former smokers from 9 European countries (Finland, France, Denmark, Germany, Italy, Spain, Sweden, Netherlands, Norway) | 45-80 | Prospective cohort consortium analysis | 0.71 (0.69-0.72) Finland to 0.81 (0.77-0.85) Germany [min-max] | Expected n = 64.1 vs. observed n = 40 Spain to expected n = 462 vs. observed n = 654 Finland [min-max] | NR | NR | NR | NR |  |
|  | Callender T et al. 2023 [54] | 40,593 PLCO Ever-Smokers | 55-74 | Analysis of data from four prospective cohorts | 0.787 (0.771-0.802) | NR | Brier score 0.0153 (0.0142-0.0164) | NR | NR | NR |  |
