# Supplementary material for: Use and impact of risk-based eligibility models in low-dose computed tomography lung cancer screening: a systematic review
Source: Public Health Rev. 2026 Jul 20;47:1609133. doi: 10.3389/phrs.2026.1609133 (PMC13430644; doi:10.3389/phrs.2026.1609133)
Supplement: Supplementary file 3 [file DataSheet1.docx]

Supplementary Material Content: Use and impact of risk-based eligibility models in low-dose computed tomography lung cancer screening: A Systematic Review

Veronika Elisabeth Mikl (VEM)^1,2,*^, Mohammad Azizzadeh (MA)^3,4^, Marie-Kathrin Breyer (MKB)^3,4,5^, Kevin ten Haaf (KTH)^6^, Valentin Ritschl (VR)^7,8^, Judit Simon (JS)^9,10^, Tanja Stamm (TS)^7,8,*^

1) Doctoral Program Public Health, Institute of Outcomes Research, Center of Medical Data Science, Medical University of Vienna, Austria

2) Roche Austria GmbH, Vienna, Austria

3) Ludwig Boltzmann Institute for Lung Health, Vienna, Austria

4) Sigmund Freud Private University, Faculty of Medicine, Vienna, Austria

5) Department of Respiratory and Pulmonary Diseases, Site Penzing of Clinic Ottakring, Vienna Healthcare Group, Vienna, Austria

6) Department of Public Health, Erasmus MC, University Medical Center Rotterdam, Rotterdam, Netherlands

7) Institute of Outcomes Research, Center of Medical Data Science, Medical University of Vienna, Austria

8) Ludwig Boltzmann Institute for Arthritis and Rehabilitation, Vienna, Austria

9) Department of Health Economics, Center for Public Health, Medical University of Vienna, Austria

10) Department of Psychiatry, University of Oxford, United Kingdom

* Corresponding authors: Tanja Stamm (TS), [tanja.stamm@meduniwien.ac.at](mailto:tanja.stamm@meduniwien.ac.at), Veronika Elisabeth Mikl (VEM), [n12410939@students.meduniwien.ac.at](mailto:n12410939@students.meduniwien.ac.at)

Contents

[1 Methods 2](#_Toc233845203)

[Supplementary File S1: TRIPOD SRMA Checklist for reporting systematic reviews of prediction model studies 2](#_Toc233845204)

[Supplementary File S2: EMBASE Search Query 4](#_Toc233845205)

[Supplementary File S3: Medline Ovid and Cochrane Central Ovid 5](#_Toc233845206)

[Supplementary File S4: Template for data extraction file displayed in rows 6](#_Toc233845207)

[2 Results 8](#_Toc233845208)

[Supplementary File S5: List of studies included to systematically review (n=46) (Risk-based Eligibility Models Review, Europe, 2025). 8](#_Toc233845209)

[Supplementary File S6: Study type categories of final assessed studies (n=46) (Risk-based Eligibility Models Review, Europe, 2025). 11](#_Toc233845210)

[Supplementary File S7: Mention frequency (%) of identified models (n=39) across assessed studies (Risk-based Eligibility Models Review, Europe, 2025). 12](#_Toc233845211)

[Supplementary File S8: Identified model types described in assessed studies listed per total count of models (Risk-based Eligibility Models Review, Europe, 2025). 13](#_Toc233845212)

[Supplementary File S9: Risk variables most mentioned in assessed models (≥10 % mentions) (Risk-based Eligibility Models Review, Europe, 2025). 15](#_Toc233845213)

[Supplementary File S10: Risk variables mentioned in assessed models (n=32, mention frequency in %) (Risk-based Eligibility Models Review, Europe, 2025). 16](#_Toc233845214)

# Methods

## Supplementary File S1: TRIPOD SRMA Checklist for reporting systematic reviews of prediction model studies

| **Section and topic** | **Item No** | **Checklist item** | **Page or Chapter** |
| --- | --- | --- | --- |
| **Title** |  |  |  |
| Title | 1 | Identify the report as a systematic review or meta-analysis (or both) of diagnostic or prognostic model studies. Specify the target population and outcome(s) predicted as relevant to the review question. | Headline |
| **Abstract** |  |  |  |
| Abstract | 2 | See the TRIPOD-SRMA Checklist for Abstracts | Abstract |
| **Introduction** |  |  |  |
| Rationale | 3 | Describe the rationale for the review in the context of existing knowledge. | Introduction |
| Objectives | 4 | Provide an explicit statement of the objective(s) being addressed with reference to: target population, index and comparator models (as relevant), outcome(s), time (prediction horizon and intended moment of using the model), and setting. | Introduction |
| **Methods** |  |  |  |
| Study eligibility criteria | 5 | Specify study characteristics used as eligibility criteria, including any prediction models of specific interest, and whether development or validation studies (or both) were eligible. | Methods, section PICO Criteria and Eligibility |
| Information sources | 6 | Specify all databases, registers, websites, organisations, reference lists and other sources searched or consulted to identify studies. Specify the date when each source was last searched or consulted. | Methods, section Search Strategy, Supplementary Files 2 and 3 |
| Search strategy | 7 | Present the full search strategies for all databases, registers and websites, including any filters and limits used. | Supplementary Files 2 and 3 |
| Study selection process | 8 | Specify the methods used to decide whether a study met the inclusion criteria of the review, including how many reviewers screened each record and each report retrieved, whether they worked independently, and if applicable, details of automation tools used in the process. | Methods, section Article selection and data extraction, Figure 1, Supplementary File 4 |
| Data collection process | 9 | Specify the methods used to collect data from study reports, including how many reviewers collected data from each report, whether they worked independently, any processes for obtaining or confirming data from study investigators, and if applicable, details of automation tools used in the process. | Methods, section Article selection and data extraction, Supplementary Table 4s |
| Data Items | 10a | List and define all items for which data were sought from each study. | Supplementary File 4 |
|  | 10b | State the model performance measures that were sought (e.g., measures of calibration, discrimination, overall model fit, clinical utility). | Table 2 |
|  | 10c | Describe how any desired but unreported data items (items 10a, 10b) were handled (e.g., contacted authors, calculated from other reported information). | Methods, section Article selection and data extraction |
| Risk of bias and applicability assessment | 11 | Specify the methods used to assess risk of bias in the included studies and their applicability to the review question. This should be done separately for each model development and validation. Include details of any tool(s) used, how many reviewers assessed each study and whether they worked independently. | NR outside research scope |
| Synthesis methods | 12a | Describe any methods for synthesising estimates of performance measures for each model. If meta-analysis was carried out, describe the methods used, including any transformations of data prior to pooling, how any heterogeneity in model performance was quantified and handled, and software package(s) used. | Methods, no meta-analysis was carried out, narrative synthesis |
|  | 12b | Describe any methods used to explore possible causes of heterogeneity in model performance (e.g., subgroup analysis, meta-regression), including whether or not they were planned. | Methods, narrative synthesis |
|  | 12c | Describe any sensitivity analyses conducted to assess robustness of the synthesised results. | NR outside research scope |
| Certainty assessment | 13 | Describe any methods used to assess certainty (or confidence) in the body of evidence for a prediction model. | Methods, section PICO Criteria and Eligibility, |
| *Page 2 / Supplementary File 1: TRIPOD SRMA Checklist for reporting systematic reviews of prediction model studies* | | | |
| **Results** |  |  |  |
| Study selection | 14 | Describe the results of the search and selection process, from the number of records identified in the search to the number of studies and models included in the review, ideally using a flow diagram. | Results, Figure 1, Supplementary File 5 |
| Study and model characteristics | 15 | Present study characteristics and model details extracted (as per Item 10a), and cite the study reports. | Results, Figure 2, Table 1, Table 2, Supplementary File 5 |
| Risk of bias and applicability | 16 | Present results of risk of bias and applicability assessment. This should be done separately for each model development and validation in each included study. | NR outside research scope |
| Results of model performance in individual studies | 17 | Present performance estimates and confidence intervals for each model and all evaluations, including whether they relate to the internal or external validation performance. If internal, give details of the method. | Results, Tables 2 and 3, Supplementary Material |
| Results of syntheses | 18a | Present the results of any synthesis of model performance, together with details of which study estimates contributed. If meta-analysis was carried out, then for each model and performance measure, present summary results, confidence/credible intervals and measures of heterogeneity. Forest plots may be useful. | Results, Tables 1, 2 and 3, Supplementary Files 5-10 |
|  | 18b | For each model, present results of all investigations of possible causes of heterogeneity in model performance. | Results, Tables 1, 2 and 3, Supplementary Files 5-10 |
|  | 18c | Present results of all sensitivity analyses conducted to assess the robustness of the synthesised results. | NR outside research scope |
| Certainty of evidence | 19 | Present any assessments of certainty (or confidence) in the body of evidence for each prediction model of interest. | Results, Figure 1, Supplementary File 5 |
| **Discussion** |  |  |  |
| Summary of evidence | 20 | Summarise the main findings including the strengths and limitations of the evidence. | Discussion, Section Main findings |
| Limitations | 21 | Discuss the strengths and limitations of the review process. | Discussion, Section Limitations |
| Implications | 22 | Discuss implications of the results in the context of other evidence and for practice, policy, and future research. | Discussion, Conclusion |
| Registration and protocol | 23a | Provide registration information for the review, including register name and registration number, or state that the review was not registered. | Methods, PROSPERO registration |
|  | 23b | Indicate where the review protocol can be accessed, or state that a protocol was not prepared. | Methods, PROSPERO registration |
|  | 23c | Describe and explain any amendments to information provided at registration or in the protocol. | Methods, PROSPERO registration |
| Support | 24 | Describe sources of financial or non-financial support for the review, and the role of the funders or sponsors in the review. | Funding sources/sponsors |
| Competing interests | 25 | Declare any competing interests of review authors. | Conflicts of interest statement |
| Availability of data, code, and other materials | 26 | Report which of the following are publicly available and where they can be found: template data collection forms; data extracted from included studies; data used for all analyses; analytic code; any other materials used in the review. | Template data collection: Supplementary File 4, data extracted from included studies: Tables 1, 2, 3, Supplementary Files 5-10, data used for all analyses: corresponding author |

## Supplementary File S2: EMBASE Search Query

Identified records with search string: 1.437

| **ID** | **EMBASE Search Query (conducted on 21.02.2025)** | **Results** |
| --- | --- | --- |
| #1 | 'lung cancer'/exp/mj | - |
| #2 | ((lung OR pulmonar*) NEAR/3 (cancer OR malignan* OR neoplasm*)):ti,kw | 238,508 |
| #3 | #1 OR #2 | 352,270 |
| #4 | 'mass screening'/mj OR 'cancer screening'/exp/mj | 68,203 |
| #5 | ((health OR mass OR population OR cancer) NEAR/3 screen*):ti,kw | 60,268 |
| #6 | #4 OR #5 | 92,143 |
| #7 | 'computer assisted tomography'/exp | 1,561,049 |
| #8 | 'computed tomography low-dose scan':ti,ab,kw OR variations of 'low-dose computed tomography' terms | 8,869 |
| #9 | 'volume computed tomography':ti,ab,kw OR 'volume ct':ti,ab,kw OR 'volumetric computed tomography':ti,ab,kw OR 'volumetric ct':ti,ab,kw | 2,900 |
| #10 | #7 OR #8 OR #9 | 1,564,098 |
| #11 | #3 AND #6 AND #10 | 4,904 |
| #12 | 'early diagnosis'/exp | 147,220 |
| #13 | (early NEAR/3 diagnos*):ti,ab,kw | 255,073 |
| #14 | 'artificial intelligence'/exp | 126,995 |
| #15 | ((machine* OR artificial* OR deep*) NEAR/3 (intelligen* OR learn*)):ti,ab,kw | 274,895 |
| #16 | 'large language model'/exp | 6,982 |
| #17 | 'large language model*':ti,ab,kw | 4,392 |
| #18 | 'prediction'/exp OR predict*:ti,kw OR forcast*:ti,kw | 1,110,332 |
| #19 | (risk* NEAR/3 (predict* OR model* OR stratif* OR base*)):ti,ab,kw,de | 361,237 |
| #20 | #12 OR #13 OR #14 OR #15 OR #16 OR #17 OR #18 OR #19 | 1,771,902 |
| #21 | #11 AND #20 | 1,437 |

## Supplementary File S3: Medline Ovid and Cochrane Central Ovid

Medline Ovid identified records with search string: 650

Cochrane Central Ovid identified records with search string: 78

| **Step** | **MEDLINE Ovid and Cochrane Central Ovid Search Query (conducted on 21.02.2025)** |
| --- | --- |
| 1. | exp *Lung Neoplasms/ |
| 2. | ((lung or pulmonar*) adj3 (cancer or malignan* or neoplasm*)).ti,kf. |
| 3. | 1 or 2 |
| 4. | exp *Mass Screening/ or exp *"Early Detection of Cancer"/ |
| 5. | ((health or mass or population or cancer) adj3 screen*).ti,kf. |
| 6. | 4 or 5 |
| 7. | exp Tomography, X-Ray Computed/ |
| 8. | (("comput* tomography" or ct) adj3 ("x ray" or "low dose" or ld or volume* or angiograph* or "cone beam")).ti,ab,kf. |
| 9. | (tomograph* adj3 "x ray" adj3 comput*).ti,ab,kf. |
| 10. | (ldct or ld-ct or uldct).ti,ab,kf. |
| 11. | 7 or 8 or 9 or 10 |
| 12. | 3 and 6 and 11 |
| 13. | early diagnosis/ |
| 14. | (early adj3 diagnos*).ti,ab,kf. |
| 15. | exp artificial intelligence/ |
| 16. | ((machine* or artificial* or deep*) adj3 (intelligen* or learn*)).ti,ab,kf. |
| 17. | "large language model*".ti,ab,kf. |
| 18. | exp Forecasting/ or predict*.ti,kf. or forcast*.ti,kf. |
| 19. | (risk* adj3 (predict* or model* or stratif* or base*)).ti,ab,kf,hw. |
| 20. | 13 or 14 or 15 or 16 or 17 or 18 or 19 |
| 21. | 12 and 20 |

## Supplementary File S4: Template for data extraction file displayed in rows

| **ID_S (ID Study)** | ID_St | Identifier (Alphanumerical / Text, e.g., "ID_St_01") |
| --- | --- | --- |
| **ID_M (ID Model)** | ID_StM | Identifier (Alphanumerical / Text, e.g., "ID_StM_01.03") |
| **Study Description** | Study (Title) | Free text |
|  | First author | Text (Categorical) |
|  | Year published | Numerical (Discrete, e.g., "2024") |
|  | Country | Categorical (Text, e.g., "EU", "US") |
|  | Study population | Numerical (Discrete integer, e.g., "240137") |
|  | Study population description | Categorical / Free Text (e.g., "Current/former") |
|  | Age lower frontier | Numerical (Discrete integer, e.g., "45") |
|  | Age upper frontier | Numerical (Discrete integer, e.g., "80") |
|  | Study type | Categorical / Text (e.g., "Prospective cohort") |
|  | Study perspective | Categorical (Dichotomous: "prospective" or "retrospective") |
| **Tool Description** | Model name | Free Text |
|  | Model group name short | Categorical (Text, e.g., "LCRAT incidence") |
|  | Model prediction lung cancer outcome | Categorical (Text: "incidence" or "mortality") |
|  | Model type | Categorical / Text (e.g., "Cox proportional hazard") |
|  | Model prediction time horizon (years) | Numerical (Discrete) / Text (e.g., "5", "1 to 5") |
| **Risk variables incorporated in model** | Age | Binary / Dichotomous (Numerical 0 or 1) |
|  | Gender | Binary / Dichotomous (Numerical 0 or 1) |
|  | Race | Binary / Dichotomous (Numerical 0 or 1) |
|  | Smoking status | Binary / Dichotomous (Numerical 0 or 1) |
|  | Former smoker | Binary / Dichotomous (Numerical 0 or 1) |
|  | Current smoker | Binary / Dichotomous (Numerical 0 or 1) |
|  | Smoking duration | Binary / Dichotomous (Numerical 0 or 1) |
|  | Smoking intensity | Binary / Dichotomous (Numerical 0 or 1) |
|  | Years since cessation | Binary / Dichotomous (Numerical 0 or 1) |
|  | Age when start smoking | Binary / Dichotomous (Numerical 0 or 1) |
|  | Pack Years | Binary / Dichotomous (Numerical 0 or 1) |
|  | Asbestos exposure | Binary / Dichotomous (Numerical 0 or 1) |
|  | Dust exposure | Binary / Dichotomous (Numerical 0 or 1) |
|  | Smoke exposure hours per day | Binary / Dichotomous (Numerical 0 or 1) |

| ***Page 2 / Supplementary File 4: Template for data extraction file displayed in rows*** | | |
| --- | --- | --- |
| **Risk variables incorporated in model** | No Hay Fever | Binary / Dichotomous (Numerical 0 or 1) |
|  | Personal history of cancer | Binary / Dichotomous (Numerical 0 or 1) |
|  | Family history of lung cancer | Binary / Dichotomous (Numerical 0 or 1) |
|  | Personal history of pneumonia | Binary / Dichotomous (Numerical 0 or 1) |
|  | Chronic bronchitis | Binary / Dichotomous (Numerical 0 or 1) |
|  | Emphysema | Binary / Dichotomous (Numerical 0 or 1) |
|  | Daily cough | Binary / Dichotomous (Numerical 0 or 1) |
|  | Education | Binary / Dichotomous (Numerical 0 or 1) |
|  | BMI | Binary / Dichotomous (Numerical 0 or 1) |
|  | COPD | Binary / Dichotomous (Numerical 0 or 1) |
|  | Diabetes | Binary / Dichotomous (Numerical 0 or 1) |
|  | Asthma | Binary / Dichotomous (Numerical 0 or 1) |
|  | Venous thromboembolism | Binary / Dichotomous (Numerical 0 or 1) |
|  | Alcohol | Binary / Dichotomous (Numerical 0 or 1) |
|  | Laboratory data | Binary / Dichotomous (Numerical 0 or 1) |
|  | Clinical variables (blood exams) | Binary / Dichotomous (Numerical 0 or 1) |
|  | Socio economic status (townsend score) | Binary / Dichotomous (Numerical 0 or 1) |
|  | Incl prev. PLCOm2012 scores and LungRADS results | Binary / Dichotomous (Numerical 0 or 1) |
| **Reporting on Outcomes** | Prediction Performance Discrimination AUC [95% CI] | Numerical (Continuous) mixed with Text (formatted with CIs or ranges) |
|  | Calibration Ability to Identify Case (ratio) | Numerical (Continuous) mixed with Text (e.g., "E/O ratio 1.05") |
|  | Detection Performance Risk-model detected LC cases (n) | Numerical (Discrete) mixed with Text |
|  | Detection Performance LC detection rate with Risk-model (%) | Numerical (Percentage) |
|  | NNS to detect 1 LC case (n) | Numerical (Discrete) / Text ranges (e.g., "588.5-763.8") |
|  | Sensitivity (%) / Specificity (%) | Numerical (Continuous / Percentages) |
|  | Incidence rate (%) | Numerical (Percentage) mixed with Text |
|  | LDCT screen rounds (n) | Numerical (Discrete) |
|  | LC deaths (n) | Numerical (Discrete) |
|  | LC deaths averted with risk model (n) | Numerical (Discrete) |
|  | LC mortality reduction (%) | Numerical (Percentage) |
|  | Cancer mortality (n) | Numerical (Discrete) |
|  | Cancer mortality rate (%) | Numerical (Percentage) |
|  | QALY accrued from screening | Numerical (Continuous / Discrete) |
|  | ICER / QALY | Numerical mixed with Text (e.g., "€72,252/QALY") |

# Results

## Supplementary File S5: List of studies included to systematically review (n=46) (Risk-based Eligibility Models Review, Europe, 2025).

| **Study Title** | **First author and year published** | **Study population** | **Study population description** | **Age of study population** | **Study type** |
| --- | --- | --- | --- | --- | --- |
| Evaluation of risk prediction models to select lung cancer screening participants in Europe: a prospective cohort consortium analysis | Feng X et al. 2024 | 240,137 | Current/former smokers from 9 European countries (Finland, France, Denmark, Germany, Italy, Spain, Sweden, Netherlands, Norway) | 45-80 | Prospective cohort consortium analysis |
| Assessing eligibility for lung cancer screening using parsimonious ensemble machine learning models: A development and validation study | Callender T et al. 2023 | 40,593 | PLCO ever-smokers | 55-74 | Analysis of data from four prospective cohorts |
| Validation of multivariable lung cancer risk prediction models for the personalized assignment of optimal screening frequency: a retrospective analysis of data from the German Lung Cancer Screening Intervention Trial (LUSI) | Maldonado S et al. 2021 | 4,052 | Ever-Smokers | 50-69 | Validation Study |
| Analysis of lung cancer risk model (PLCOM2012 and LLPv2) performance in a community-based lung cancer screening programme | Lebrett M et al. 2020 | 1,429 | Ever-Smokers Manchester Lung Health Check (LHC) pilot | 55-74 | Comparison Study |
| Risk prediction models for selection of lung cancer screening candidates: A retrospective validation study | Ten Haaf K et al. 2017 | 134,124 | NLST & PLCO ever-smokers | 57-69 | Retrospective validation study |
| Development and Validation of Risk Models to Select Ever-Smokers for CT Lung Cancer Screening | Katki H et al. 2016 | 132,160 | PLCO & NLST ever-smokers | 55-74 | Development and Validitation Study |
| Prospective Evaluation of Lung Cancer Screening Eligibility Criteria and Lung Cancer Detection in the Yorkshire Lung Screening Trial | Gabe R et al. 2024 | 7,826 | Ever-smokers in Yorkshire, UK | 55-80 | Prospective cohort study (randomized controlled trial with community-based LDCT-LCS) |
| Swiss Pilot Low-Dose CT Lung Cancer Screening Study: First Baseline Screening Results | Jungblut L et al. 2023 | 112 | Asymptomatic participants at high risk for lung cancer (55-74 years) | 55-74 | Prospetcive Pilot Study |
| Toward a Computable Phenotype for Determining Eligibility of Lung Cancer Screening Using Electronic Health Records | Yang S et al. 2025 | 5,778 | Individuals from University of Florida Health Integrated Data Repository (2012–2022 LDCT recipients) | 50-80 | Original quantitative evaluation study |
| Cost-effectiveness of a machine learning risk prediction model (LungFlag) in the selection of high-risk individuals for non-small cell lung cancer screening in Spain | Trujillo J C et al. 2025 | 3,835,128 | Individuals meeting USPSTF 2013 criteria in Spain (adults aged 55-80 with ≥30 pack-year smoking history) | 55-80 | Cost-effectiveness analysis using decision-tree + Markov model |
| Risk-based lung cancer screening performance in a universal healthcare setting. | Tammemägi M et al. 2024 | 7,768 | High-risk individuals in Ontario, Canada | 55-74 | real world case study, multi-center lung cancer screening pilot |
| Cost-Effectiveness of risk-based low-dose computed tomography screening for lung cancer in Switzerland | Tomonaga Y et al. 2023 | 10 Mio | Birth cohort 1940-1979 (MISCAN Model), ever-smokers 10-40 pack years | 50-85 | Microsimulation-based cost-effectiveness and budget impact analysis based on MISCAN model |
| Results of a pilot risk‑based lung cancer screening study: outcomes and comparisons to a Medicare eligible cohort | Hirsch E et al. 2023 | 48 | Ever-Smokers | 40-82 | Prospective Pilot Study (NCT03683940) |
| Safety net hospital risk model demonstrates stronger, population-specific applicability in characterizing lung cancer risk | Rodriguez A et al. 2024 | 896 | High-risk individuals from Boston Medical Center (BMC) database of patients who received LC screening with LDCT between 2015 to 2019 | 57-70 | Retrospective cross-sectional study |
| Performance of various risk prediction models in a large lung cancer screening cohort in Gdańsk, Poland—a comparative study | Ostrowski M et al. 2020 | 6,631 | Ever-Smokers, ≥30 pack-years smoking history | 50-79 | comparative study with cohort data MOLTEST BIS programme |
| OWL: an optimized and independently validated machine learning prediction model for lung cancer screening based on the UK Biobank, PLCO, and NLST populations | Pan Z et al. 2023 | 323,344 | General population (UK Biobank, UKB) | 37-73 | Development and validation study |
| Risk Model–Based Lung Cancer Screening A Cost-Effectiveness Analysis | Toumazis I et al. 2023 | 1,000,000 | 1960 US birth cohort | 45-90 | Comparative modelling analysis based on four microsimulation models (CISNET) |

| *Page 2/3 - Supplementary File 5: List of 46 studies included to systematically review (Europe, 2025)* | | | | | |
| --- | --- | --- | --- | --- | --- |
| **Study Title** | **First author and year published** | **Study population** | **Study population description** | **Age of study population** | **Study type** |
| Comparison of discrimination performance of 11 lung cancer risk models for predicting lung cancer in a prospective cohort of screening-age adults from Germany followed over 17 years | Bhardwaj M et al. 2022 | 9,407 | Population from ESTHER cohort study | 50-75 | Comparative evaluation study |
| Health utilities for participants in a population-based sample who meet eligibility criteria for lung cancer screening. | Ngo PJ et al. 2022 | 19,991 | Ever-Smokers (current/former) from NSW 45 and Up Study cohort, Australia | 50-80 | Quantitative evaluation using population-based cohort data |
| Lung cancer screening use and implications of varying eligibility criteria by race and ethnicity: 2019 Behavioral Risk Factor Surveillance System data. | Williams RM et al. 2022 | 41,544 | Current/former smokers | 50-80 | Cross-sectional analysis of 2019 Behavioral Risk Factor Surveillance System (BRFSS) data |
| A Risk-Based Framework for Assessing Real- Time Lung Cancer Screening Eligibility That Incorporates Life Expectancy and Past Screening Findings | Toumazis I et al. 2020 |  | Subpopulation of Ever-Smokers | 50-80 | Original quantitative evaluation study using partially observable Markov decision process (POMDP) |
| Evaluation of the Benefits and Harms of Lung Cancer Screening With Low-Dose Computed Tomography | Meza R et al. 2021 | 1,000,000 | 1950 and 1960 US birth cohort | 45-90 | Comparative simulation modelling study based on four microsimulation models (CISNET) |
| Performance of Risk Factor-Based Guidelines and Model-Based Chest CT Lung Cancer Screening in World Trade Center-Exposed Fire Department Rescue/Recovery Workers | Cleven K et al. 2021 | 3,953 | Ever-Smokers, FDNY-WTC exposed rescue and recovery workers (firefighters/EMS) | 50-80 | Retrospective analysis |
| Liverpool Lung Project lung cancer risk stratification model: calibration and prospective validation | Field J K et al. 2021 | 75,958 | Risk population | 50-79 | Case-control and prospective cohort designs |
| Organized Lung Cancer Screening Pilot: Informing a Province-Wide Program in Ontario, Canada. | Darling G et al. 2021 | 4,205 | Individuals aged 55-74, current/ex-smokers (≥20 pack-years), excluding those with diagnosed lung cancer or under nodule surveillance | 55-74 | Original quantitative evaluation study |
| Assessing Different Approaches to Leveraging Historical Smoking Exposure Data to Better Select Lung Cancer Screening Candidates: A Retrospective Validation Study | Kats et al. 2021 | 37,327 | Ever-Smokers | 55-74 | Retrospective cohort validation study based on EHR |
| Baseline Results of the West London lung cancer screening pilot study - Impact of mobile scanners and dual risk model utilisation. | Bartlett E et al. 2020 | 8,366 | Ever-smokers in West London, UK | 60-75 | Prospective observational pilot study |
| Risk prediction models versus simplified selection criteria to determine eligibility for lung cancer screening: an analysis of German federal-wide survey and incidence data | Hüsing A et al. 2020 | 16 Mio. projected | 16 Mio. projected from 14,834 Ever-Smokers of German population study GEDA 2008-2013 and 20.700 Ever-Smokers from German EPIC cohort (EPIC-D) | 50-79 | Retrospective analysis |
| Development and Validation of a Multivariable Lung Cancer Risk Prediction Model That Includes Low-Dose Computed Tomography Screening Results: A Secondary Analysis of Data From the National Lung Screening Trial | Tammemägi M et al. 2019 | 22,229 | Ever-Smokers, NLST participants (LSS and ACRIN subsets) | 57-65 | Secondary analysis of NLST RCT data |
| Outcomes of Long-term Interval Rescreening With Low-Dose Computed Tomography for Lung Cancer in Different Risk Cohorts. | Aggarwal R et al. 2019 | 1,261 | Adults aged ≥50 with ≥10 pack-year smoking history, no prior cancer (except nonmelanotic skin), negative baseline LDCT scans | 50-74 | Prospective single-arm cohort study |
| ‘Reduced’ HUNT model outperforms NLST and NELSON study criteria in predicting lung cancer in the Danish screening trial | Roe O D et al. 2019 | 4,051 | DLST cohort participants | 50-70 | Retrospective analysis of DLCST cohort data |
| Implications of Nine Risk Prediction Models for Selecting Ever-Smokers for Computed Tomography Lung Cancer Screening | Katki H et al. 2018 | 409,726 | Ever-smokers from National Institutes of Health–AARP Diet and Health Study (NIH AARP) and ever-smokers from CPS-II (Cancer Prevention Study II) Nutrition Survey cohort | 50-80 | Comparison and Validation Study |
| Participant selection for lung cancer screening by risk modelling (the Pan-Canadian Early Detection of Lung Cancer [PanCan] study): a single-arm, prospective study | Tammemägi M et al. 2017 | 7,044 | Ever-smokers aged 50–75 years without lung cancer history, recruited across eight Canadian centers | 50-75 | Singe-arm, prospective study |
| Identifying high risk individuals for targeted lung cancer screening: Independent validation of the PLCO m2012 risk prediction tool | Weber M et al. 2017 | 5,882 | Australian ever-smokers from the 45 and Up Study cohort | 45-100 | Population-based cohort study |
| *Page 3/3 - Supplementary File 5: List of 46 studies included to systematically review (Europe, 2025)* | | | | | |
| **Study Title** | **First author and year published** | **Study population** | **Study population description** | **Age of study population** | **Study type** |
| A simple model for predicting lung cancer occurrence in a lung cancer screening program: The Pittsburgh Predictor | Wilson D et al. 2015 | 57,108 | Current and former smokers (PLuSS and NLST | 50-79 | Original quantitative evaluation of risk prediction models |
| Selection Criteria for Lung-Cancer Screening | Tammemägi M et al. 2013 | 133,580 | PLCO and NLST ever-smokers | 55-74 | Retrospective modeling study |
| Predictive Accuracy of the Liverpool Lung Project Risk Model for Stratifying Patients for Computed Tomography Screening for Lung Cancer | Raji O et al. 2012 | 2,922 | EUELC (European), Harvard (North American), and LLPC (UK) study | 40-79 | Case-control & prospective cohort |
| Lung Cancer Risk Prediction to Select Smokers for Screening CT—a Model Based on the Italian COSMOS Trial | Maisonneuve P et al. 2023 | 5,203 | Asymptomatic individuals aged ≥50, heavy smokers (≥20 pack-years) | 50-84 | Prospective cohort study (based on the COSMOS trial) |
| Variations in Lung Cancer Risk Among Smokers | Bach P et al. 2003 | 18,314 | Heavy smoker and asbestos cohort | 44-75 | modelling study based on CARET cohort study |
| Targeted lung cancer screening in the age of immunotherapies and targeted therapies - an economic evaluation for Australia | Roseleur J et al. 2024 | 6,700,000 | Birth cohort 1945-1969 (5 cohorts) | 54-78 | Microsimulation-based cost-effectiveness and budget impact analysis based on MISCAN model (5 cohorts) |
| Predicting the future risk of lung cancer: development, and internal and external validation of the CanPredict (lung) model in 19·67 million people and evaluation of model performance against seven other risk prediction models | Liao W et al. 2023 | 19,670,000 | Asymptomatic adults from English primary care databases (QResearch & CPRD) | 25-84 | Retrospective population-based cohort study |
| Targeting of Low-Dose CT Screening According to the Risk of Lung-Cancer Death | Kovalchik S et al. 2013 | 53,158 | LDCT Group & Radiography Group | 55-74 | Modeling Study |
| Population-based systematic enrolment of individuals ensures high lung cancer screening uptake | Laisaar T et al. 2025 | 26,759 | Individuals aged 55–74 years who had ever smoked | 55-74 | Estonian regional LCS pilot project |
| Evaluation of the Lung Cancer Risks at Which to Screen Ever- and Never-Smokers: Screening Rules Applied to the PLCO and NLST Cohorts (incl. Correction Table 1) | Tammemägi M et al. 2014 | 208,352 | PLCO & NLST ever-smokers | 55-74 | Evaluation study |
| Machine Learning for Early Lung Cancer Identification Using Routine Clinical and Laboratory Data | Gould M et al. 2021 | 196,102 | NSCLC case patients | 45-90 | Retrospective cohort study, Kaiser Permanente Southern California (KPSC) |
| The LLP risk model: an individual risk prediction model for lung cancer | Cassidy A et al. 2007 | 1,736 | Residents of the Liverpool area, incident cases of histologically or cytologically confirmed lung cancer were between 20 and 80 years of age | 57-75 | Case-control study |

## Supplementary File S6: Study type categories of final assessed studies (n=46) (Risk-based Eligibility Models Review, Europe, 2025).


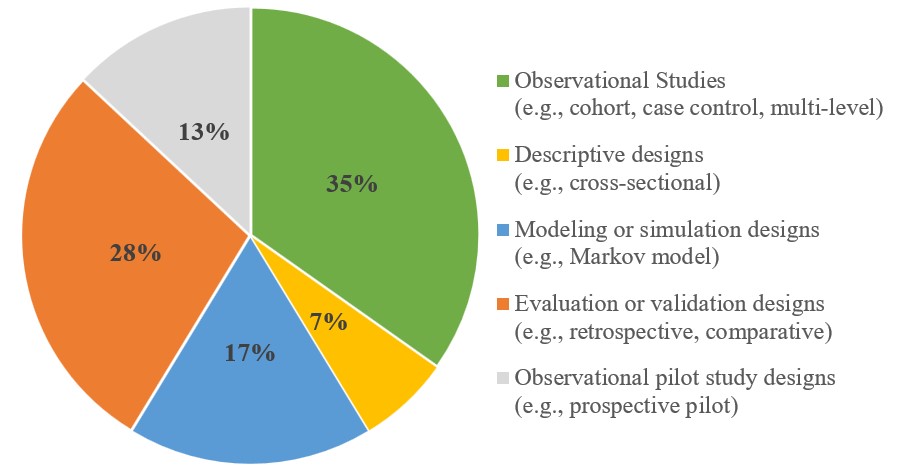


## Supplementary File S7: Mention frequency (%) of identified models (n=39) across assessed studies (Risk-based Eligibility Models Review, Europe, 2025).


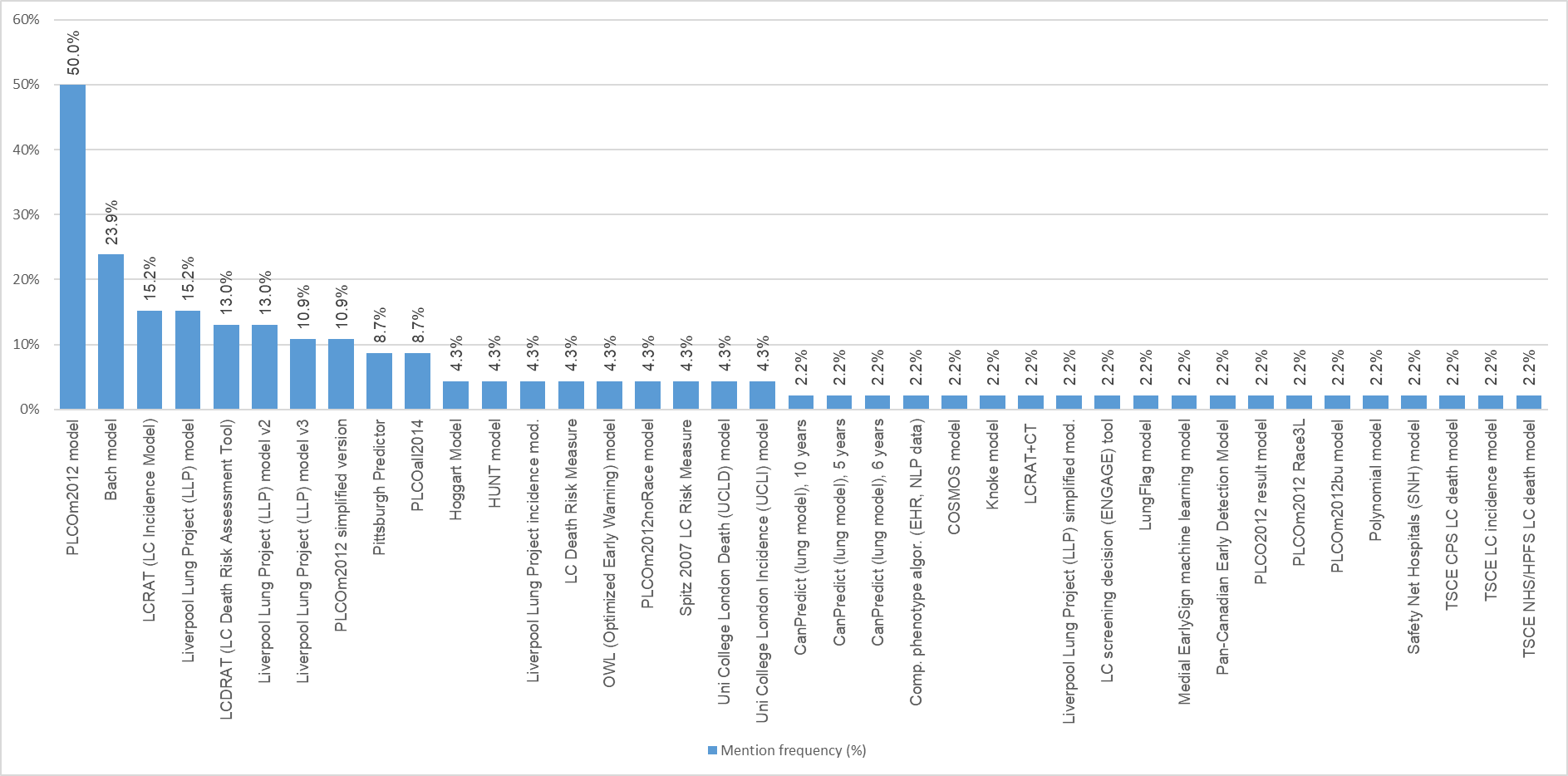


## Supplementary File S8: Identified model types described in assessed studies listed per total count of models (Risk-based Eligibility Models Review, Europe, 2025).

| **Type Family** | **n** | **Model type described in assessed studies** | **Name of LC Risk-Prediction Model in assessed studies** |
| --- | --- | --- | --- |
| **A) Statistical approaches** including classical regression and survival analysis models | 10 | Logistic-regression model | PLCOm2012 model |
|  |  |  | PLCOm2012 model simplified version |
|  |  |  | PLCOall2014 |
|  |  |  | PLCOm2012noRace model |
|  |  |  | Safety Net Hospitals (SNH) model |
|  |  |  | Polynomial model |
|  |  |  | PLCOm2012bu model |
|  |  |  | PLCOm2012 Race3L |
|  |  |  | PLCO2012 result model |
|  |  |  | Pan-Canadian Early Detection of LC (PanCan) Model |
|  | 5 | Logistic-regression model for relative risks; adjustment of intercept to match Liverpool age-, sex, smoking-status incidence rates | Liverpool Lung Project (LLP) model |
|  |  |  | Liverpool Lung Project (LLP) model version 2 |
|  |  |  | Liverpool Lung Project (LLP) model version 3 |
|  |  |  | Liverpool Lung Project (LLPi) incidence model |
|  |  |  | Liverpool Lung Project (LLP) model simplified version |
|  | 5 | Cox proportional hazards model | COSMOS model |
|  |  |  | CanPredict (lung model), 6 years |
|  |  |  | CanPredict (lung model), 5 years |
|  |  |  | CanPredict (lung model), 10 years |
|  |  |  | LCRAT+CT |
|  | 2 | Cox proportional hazards model with non-parametric baseline hazards in prospective cohort | LCRAT (Lung Cancer Incidence Model) |
|  |  |  | LCDRAT (Lung Cancer Death Risk Assessment Tool) |
|  | 1 | Cox proportional hazards model; recursive estimation for projections beyond 1 year | Bach model |
|  | 1 | 4-factor logistic regression model | Pittsburgh Predictor |
|  | 1 | Weibull logistic-regression model in prospective cohort with stratification by status, age-initiated smoking and quit years | Hoggart Model |
|  | 1 | Multivariable regression model | LC Death Risk Measure |
|  | 1 | Multivariable Cox regression model with non-linear transformations | HUNT model (Norwegian Nord-Trøndelag Health Study model) |
|  | 1 | Two-parameter Poisson regression model | Knoke model |
| *Page 2/2 - Supplementary File 8: Identified model types described in assessed studies listed per total count of models (Europe, 2025)* | | | |
| **Type Family** | **n** | **Model type described in assessed studies** | **Name of LC Risk-Prediction Model in assessed studies** |
| **B) Computational approaches** including stochastic modeling, Markov decision processes, and algorithmic phenotyping | 3 | Stochastic representation of the cell events | Two-Stage Clonal Expansion (TSCE) NHS/HPFS LC death model |
|  |  |  | Two-Stage Clonal Expansion (TSCE) LC incidence model |
|  |  |  | Two-Stage Clonal Expansion (TSCE) CPS LC death model |
|  | 1 | Logistic recursively cycling model for relative risks; attributable risk method applied to SEER incidence and mortality rates to obtain baseline rate | Spitz 2007 Lung Cancer Risk Measure |
|  | 1 | Partially observable Markov decision process (POMDP) | LC screening decision (ENGAGE) tool |
|  | 1 | Computable phenotype algorithm | Computable phenotype (CP) algorithms for LCS eligibility, Rule-based algorithm combining structured EHR data and NLP-processed unstructured data |
| **C) Machine learning approaches**  including ensemble methods and algorithms like XGBoost | 2 | Machine learning model (ensemble) | University College London Incidence (UCLI) model |
|  |  |  | University College London Death (UCLD) model |
|  | 1 | XGBoost machine learning algorithm (ensemble) | OWL (Optimized Early Warning Model for Lung Cancer Risk) model |
|  | 1 | XGBoost (Extreme Gradient Boosting) | Medial EarlySign (MES) machine learning model |
|  | 1 | Machine learning algorithm | LungFlag model |

## Supplementary File S9: Risk variables most mentioned in assessed models (≥10 % mentions) (Risk-based Eligibility Models Review, Europe, 2025).


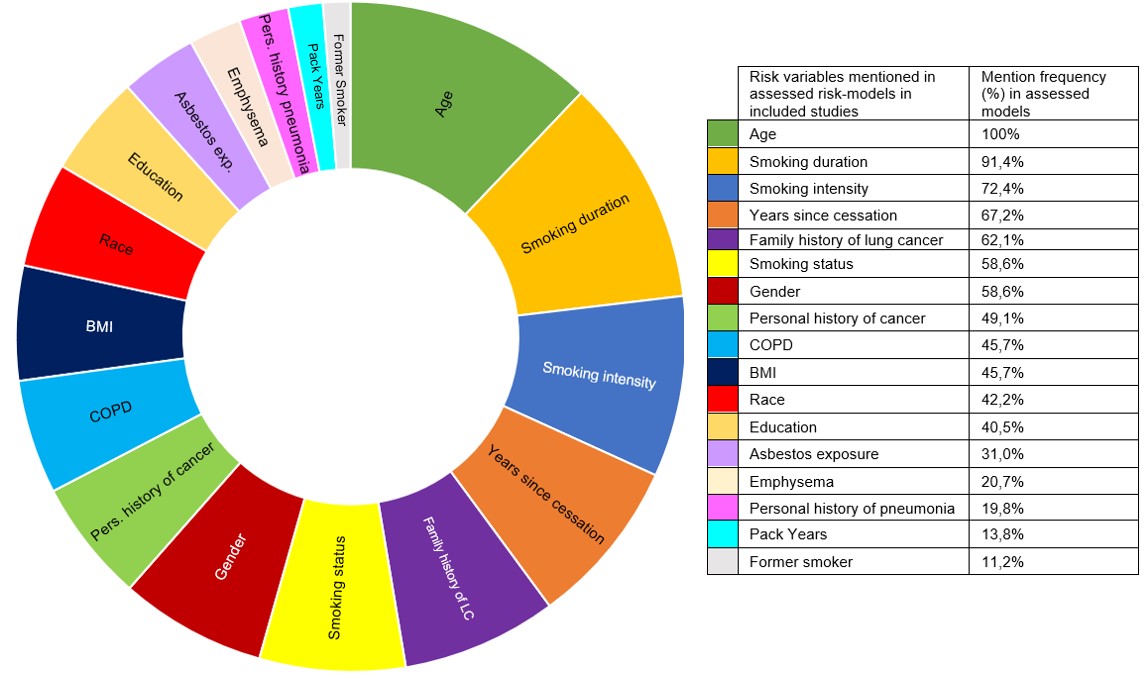


## Supplementary File S10: Risk variables mentioned in assessed models (n=32, mention frequency in %) (Risk-based Eligibility Models Review, Europe, 2025).

| **Number** | **Risk variables mentioned in assessed risk-models** | **Frequency (%)** |
| --- | --- | --- |
| 1 | Age | 100 % |
| 2 | Smoking duration | 91,4 % |
| 3 | Smoking intensity | 72,4 % |
| 4 | Years since cessation | 67,2 % |
| 5 | Family history of lung cancer | 62,1 % |
| 7 | Smoking status | 58,6 % |
| 6 | Gender | 58,6 % |
| 8 | Personal history of cancer | 49,1 % |
| 9 | BMI | 45,7 % |
| 10 | COPD | 45,7 % |
| 11 | Race | 42,2 % |
| 12 | Education | 40,5 % |
| 13 | Asbestos exposure | 31,0 % |
| 14 | Emphysema | 20,7 % |
| 15 | Personal history of pneumonia | 19,8 % |
| 16 | Pack Years | 13,8 % |
| 17 | Former smoker | 11,2 % |
| 18 | Current smoker | 9,5 % |
| 19 | No Hay Fever | 4,3 % |
| 20 | Alcohol | 3,4 % |
| 21 | Daily cough | 3,4 % |
| 22 | Dust exposure | 3,4 % |
| 23 | Smoke exposure hours per day | 3,4 % |
| 24 | Venous thromboembolism | 3,4 % |
| 25 | Asthma | 2,6 % |
| 26 | Age when start smoking | 2,6 % |
| 27 | Socio economic status (townsend score) | 2,6 % |
| 28 | Chronic bronchitis | 1,7 % |
| 29 | Diabetes | 0,9 % |
| 30 | Clinical variables (blood exams) | 0,9 % |
| 31 | Prev. PLCOm2012 scores and LungRADS results | 0,9 % |
| 32 | Laboratory data (without further details mentioned) | 0,9 % |
